# Interleukin-6 and Interferon-α Signaling *via* JAK1–STAT Differentially Regulate Oncolytic versus Cytoprotective Antiviral States

**DOI:** 10.3389/fimmu.2018.00094

**Published:** 2018-01-30

**Authors:** Oded Danziger, Tal Pupko, Eran Bacharach, Marcelo Ehrlich

**Affiliations:** ^1^Department of Cell Research and Immunology, George S. Wise Faculty of Life Sciences, Tel Aviv University, Tel Aviv, Israel

**Keywords:** interferon, interleukin-6, epizootic hemorrhagic disease virus, viral oncolysis, prostate cancer, JAK1, STAT1, STAT3

## Abstract

Malignancy-induced alterations to cytokine signaling in tumor cells differentially regulate their interactions with the immune system and oncolytic viruses. The abundance of inflammatory cytokines in the tumor microenvironment suggests that such signaling plays key roles in tumor development and therapy efficacy. The JAK–STAT axis transduces signals of interleukin-6 (IL-6) and interferons (IFNs), mediates antiviral responses, and is frequently altered in prostate cancer (PCa) cells. However, how activation of JAK–STAT signaling with different cytokines regulates interactions between oncolytic viruses and PCa cells is not known. Here, we employ LNCaP PCa cells, expressing (or not) JAK1, activated (or not) with IFNs (α or γ) or IL-6, and infected with RNA viruses of different oncolytic potential (EHDV-TAU, hMPV-GFP, or HIV-GFP) to address this matter. We show that in JAK1-expressing cells, IL-6 sensitized PCa cells to viral cell death in the presence or absence of productive infection, with dependence on virus employed. Contrastingly, IFNα induced a cytoprotective antiviral state. Biochemical and genetic (knockout) analyses revealed dependency of antiviral state or cytoprotection on STAT1 or STAT2 activation, respectively. In IL-6-treated cells, STAT3 expression was required for anti-proliferative signaling. Quantitative proteomics (SILAC) revealed a core repertoire of antiviral IFN-stimulated genes, induced by IL-6 or IFNs. Oncolysis in the absence of productive infection, induced by IL-6, correlated with reduction in regulators of cell cycle and metabolism. These results call for matching the viral features of the oncolytic agent, the malignancy-induced genetic-epigenetic alterations to JAK/STAT signaling and the cytokine composition of the tumor microenvironment for efficient oncolytic virotherapy.

## Introduction

The Janus family of evolutionary-conserved non-receptor tyrosine kinases comprises four members in mammals: JAK1, JAK2, JAK3, and TYK2 (1). These large proteins contain four domains: (1) a kinase domain; (2) an enzymatically inactive pseudo-kinase domain that modulates the kinase activity; and (3–4) the SH2 and FERM domains that mediate protein–protein interactions (1). All Janus kinases (JAKs) interact with cognate cytokine receptors and transduce signals involved in immunity and inflammation (2, 3). In spite of this functional similarity, the notion of non-redundant functions for the different JAK proteins is supported by the differences in phenotype of JAK1-3/TYK2-knockout mice (1). In cancer, JAK signaling plays a dual role, as exacerbated signaling is typical of certain types of leukemia, while many solid tumors, including prostate cancer (PCa), are characterized by defects in interferon-induced JAK/STAT signaling.

Prostate cancer cells account for the highest number of cancer diagnoses and the second-highest number of cancer-related cell deaths among American men (4). While chemical castration inhibits tumor growth at initial stages, metastatic PCa is currently incurable (5, 6). Prostate tumorigenesis is supported by cell autonomous mechanisms including point mutations, chromosomal aberrations (7–9), and epigenetic silencing of tumor-suppressor genes (10, 11). In conjuncture with an inflammatory microenvironment, these molecular aberrations alter the activation state and function of a plethora of signal transduction pathways, including the JAK—signal transducer and signal transducer and activator of transcription (STAT) pathway (12). We and others have previously shown that a subset of PCa cells, and the LNCaP PCa cell line in particular, are defective in JAK1 expression (13–15). Notably, LNCaP cells express JAK2 and TYK2, but not JAK3, suggesting that multiple cytokine stimuli can be differently interpreted by these cells due to their expression pattern of JAK (16). Importantly, we have demonstrated that lack of JAK1 expression is associated with interferon-insensitivity and with hypersusceptibility of these cells to viral infections (15). In addition to interferon signaling, JAK1 mediates signaling by families of receptors that share the γc or gp130 subunits. Thus, differences in JAK1 expression may result in an altered cellular response to multiple different stimuli. One of the important signals that is mediated by JAK1 is that of interleukin-6 (IL-6) and its cognate receptors (1, 17).

Interleukin-6 is a pleiotropic cytokine that plays key roles in infection and immunity *via* the regulation of the acute-phase response, the expansion and activation of T cells, and the differentiation of B cells (18). In addition to immunity-related functions, IL-6 stimuli modulate basic biological processes including lipid metabolism and mitochondrial activities, resulting in regulation of the neuroendocrine and vascular systems and behavior (18). Canonical signaling by IL-6 involves ligand binding by membrane-bound or soluble IL-6 receptors (IL-6R), followed by their recruitment into a complex with the 130-kDa signal transducing β-receptor subunit (gp130) (19–21). In addition to transduction of signals *via* the JAK/STAT pathway, IL-6 signals are also mediated *via* the MAPK and PI3K intracellular pathways (17). IL-6 in general, and IL-6 transsignaling (signaling mediated by the soluble IL-6R) in particular, are known to play deleterious roles in cancer (20, 21). In PCa patients, IL-6 serum levels correlate with cancer progression and metastatic disease (22–25). Conversely, levels of soluble gp130, predicted to inhibit IL-6 transsignaling (26), are also positively correlated with PCa progression (27), suggesting a complex role for IL-6 in prostate malignancy. Cellular models of PCa also exhibit diverse IL-6-signaling-related phenomena, including growth inhibition, growth stimulation, neuroendocrine transdifferentiation, or epithelial-to-mesenchymal transition (28–38). The effects of IL-6 on PCa cells depend on the length of the stimulation and on androgen-dependency of the cells in question (28, 33, 37). The roles of IL-6 in mouse models of PCa seem similarly complex, as it either inhibits growth or promotes survival of xenografts (36, 39, 40) or regulates transdifferentiation in a model of autochthonous PCa (41).

LNCaP cells are a broadly employed model of hormone-responsive PCa cells (42) which are sensitive to IL-6-induced transdifferentiation (16, 29–31, 34, 35). LNCaP cells express both the 80-kDa (transmembrane) and the 55-kDa (soluble) isoforms of the IL-6R, in addition to TYK2 and JAK2, suggesting that they can perform both canonical and trans-IL-6 signaling (16). Importantly, in spite of JAK1 being a central mediator of IL-6 signaling (43), the lack of JAK1 expression in LNCaP cells [due to genetic mutations and epigenetic silencing (13–15)] was not experimentally addressed in the context of IL-6 signaling. Additionally, the lack of JAK1 expression renders these cells interferon-insensitive and susceptible to infection with different classes of oncolytic viruses (15, 44–47).

In this work, we employed wt and JAK1-expressing LNCaP cells to compare and contrast IL-6 and IFN signaling, in the context of infection with viruses of different oncolytic potential. To obtain a highly oncolytic virus, we made use of the Ibaraki (IBA) strain of the Epizootic Hemorrhagic Disease virus (EHDV2-IBA), which naturally infects ruminants, is cytolytic, and induces apoptosis, necroptosis, autophagy, and cell stress (48). Through serial passaging of EHDV2-IBA in LNCaP cells, we obtained viruses exhibiting six orders of magnitude fold increase in titer, relative to the parental virus. We isolated one such adapted strain and named it “EHDV-TAU.” In accord with its potential to function as an oncolytic reagent, EHDV-TAU infection was greatly restricted in untransformed interferon-responsive human cells (15). As a virus with mild oncolytic potential, we employed the human metapneumovirus (hMPV), a respiratory pathogen and a member of the Paramyxoviridae family. We constructed a replication-competent derivative of this virus that expresses GFP [hMPV-GFP (49)] and have recently shown that it productively infects LNCaP cells, albeit with limited cytolytic effect (15). As a virus that is predicted to be devoid of cytolytic activity in this system, we employed a lenti vector that expresses GFP (HIV-GFP) (50).

We show that in JAK1-expressing cells, IL-6 sensitized PCa cells to virally induced cell death. For EHDV-TAU, IL-6 induced oncolysis in the absence of productive infection, while this cytokine augmented the mild cytolytic activity of hMPV-GFP. These effects of IL-6 were in contrast to the cytoprotective antiviral state induced by IFNα. These contrasting outcomes correlated with differing profile of activation of STAT proteins and with specific changes to the cellular proteome.

## Materials and Methods

### Cell Culture and Viruses

The identity of LNCaP (ATCC^®^ CRL-1740™) and castration resistant DU145 (ATCC^®^ HTB-81™) PCa cells was confirmed by Short Tandem Repeat (STR) analysis at the biomedical core facility at the genomic center (Technion, Israel). Baby Hamster Kidney cells (BHK-21, ATCC CCL-10) were employed for plaque assays (see details of this assay below). LNCaP cells were cultured in Roswell Park Memorial Institute medium (RPMI 1640) supplemented with 2-mM l-glutamine, 10-mM HEPES, and 1-mM sodium pyruvate. BHK-21 cells were cultured in Modified Eagle’s Medium (MEM), supplemented with 2-mM l-glutamine. DU145 cells were grown in Dulbecco’s Modified Eagle’s Medium (DMEM). Culture media were supplemented with 10% Fetal Calf Serum (FCS) and penicillin–streptomycin–neomycin solution (culture reagents are from Beit Haemek Biological Industries). Cultures were grown at 37°C and 5% CO_2_. Generation, propagation, and purification of EHDV-TAU and hMPV-GFP were previously described (15, 48, 49). GFP-expressing, VSV-G-pseudotyped-HIV-based vector (HIV-GFP) was previously described (50, 51). Moreover, 14 h prior to infection with EHDV-TAU, hMPV-GFP, or HIV-GFP, cells were treated with the indicated combination of cytokines and chemicals. Handling of all viruses was according to safety regulations of the Tel Aviv University. Infections were carried for the indicated time in the presence of these reagents. hMPV-GFP infections were carried for 6 h in infection media (RPMI supplemented with 3% FCS, 5-mM glutamine and penicillin–streptomycin and 0.25-mg/mL trypsin), after which infection media were replaced with RPMI supplemented with 10% FCS containing the indicated combination of cytokines and chemicals.

### Immunoblotting and Antibodies

Immunoblotting was done as previously described (15) employing the following antibodies and dilutions: anti-NS3 antibodies were described in Ref. (48), rabbit anti-phospho-Tyr701-STAT1 (cat. #9167; 1:1,000), rabbit anti-STAT1 (cat. #9172; 1:1,000), rabbit anti-phospho-Tyr690-STAT2 (cat. #88410; 1:1,000), rabbit anti-STAT2 (cat. #72604; 1:1,000) rabbit anti-phospho-Tyr705-Stat3 (cat. #9145; 1:1,000), mouse anti-STAT3 (cat. #9139; 1:1,000), and rabbit anti-SOCS3 (cat. #2923; 1:1,000), (all from cell signaling); mouse anti-phospho-ERK1/2 (Sigma-Aldrich, cat. #M8159; 1:500), rabbit anti-ERK1/2 (Santa-Cruz Biotechnology, cat. #sc-154; 1:10,000), rabbit-anti-JAK1 (Santa-Cruz Biotechnology, cat. #sc-277; 1:400), mouse anti-Actin (MP Biomedicals, cat. #69100; 1:10,000), rabbit anti-GAPDH (Abcam, cat. #ab8245; 1:5,000), mouse anti-GFP (MBL, cat. #M048-3; 1:1,000), and HRP-conjugated secondary antibodies (Jackson ImmunoResearch Laboratories, cat. #115035003; 1:15,000).

### Cytokines and Reagents

Reagents were employed at the following final concentrations: human IFNα (PBL-assay science, cat. #111051), 200 U/mL; human IL-6 (PeproTech, cat. #200-06), 5 ng/mL unless stated otherwise; human IFNγ (PeproTech, cat #300-02), 25 ng/mL; Quinolyl-valyl-O-methylaspartyl-[-2,6-difluorophenoxy]-methyl ketone (Q-VD-OPh; ApexBio Technology, cat. #A1901), 20 µM; JAK inhibitor (Baricitinib, BioVision, cat. #2842), 0.5 µM.

### Plaque Assay, Trypan Blue Exclusion Assay, and qRT-PCR

The plaque assay and the trypan blue exclusion assay were conducted as follows (15, 48). For qRT-PCR, the following primers were used: JAK1fw: 5′GGAAGTGCGCTTCTCTG′3, JAK1rev 5′CTGCATTTATTCAGCTGTCC′3, IFIT5fw 5′GCACTTTAAACAAGCTCCTCCTA′3, IFIT5rev 5′CCAAGTTTGAGGAACAATGCT′3, IRF7fw 5′CCCAGCAGGTAGCATTCCC′3, IRF7rev 5′GCAGCAGTTCCTCCGTGTAG′3, SOCS3fw 5′GGAGACTTCGATTCGGGACC′3, SOCS3rev 5′GAAACTTGCTGTGGGTGACC′3, p21fw 5′CTGCCCAAGCTCTACCTTCC′3, p21rev 5′CAGGTCCACATGGTCTTCCT′3, IRF9fw 5′TCCTCCAGAGCCAGACTACT′3, IRF9rev 5′CAATCCAGGCTTTGCACCTG′3 RIG-Ifw 5′GACCCTGGACCCTACCTACA′3, RIG-Irev 5′CTCCATTGGGCCCTTGTTGT′3, GAPDHfw 5′AGCCACATCGCTCAGACAC′3, and GAPDHrev 5′GCCCAATACGACCAAATCC′3.

### Cell Proliferation Assay

To assess proliferation, cells were plated for 72 h in 96-well plate (5,000 cells/well; six repetitions for each time point/condition). Cells were fixed (2 h) every 24 h with 4% formaldehyde, stained with 0.5% methylene blue in 0.1-M sodium borate, and extracted with 0.1-M HCl. Absorbance was measured at 595 nm.

### Fluorescence-Activated Cell Sorting (FACS) Analysis

LNCaP-JAK1 cells (see Results), pretreated or not with 5 ng/mL IL-6 (16 h) and infected (48 h) with GFP-expressing VSV-G-pseudotyped-HIV (HIV-GFP) particles (50), were fixed with 2% paraformaldehyde and analyzed by FACS (Becton Dickinson) for GFP fluorescence. Uninfected LNCaP-JAK1 cells were used to determine background autofluorescence. Data were analyzed with the FlowJo software (BD Biosciences).

### Cell Cycle Analysis

LNCaP-JAK1 cells, pretreated (or not) with IL-6 (5 ng/mL, 16 h) and infected (or not) with EHDV-TAU (48 h, moi = 0.5), were trypsinized, washed with cold phosphate-buffered saline (PBS), and fixed in ice-cold methanol (1 mL, 20 min, −20°C). RPMI (supplemented with 10% FCS, 10 mL) was added postfixation, after which cells were pelleted, washed twice with cold PBS and resuspended in PBS supplemented with RNase A (20 µg/mL, 30 min). Following additional pelleting, cells were resuspended in propidium iodide (PI) solution (50 µg/mL in PBS) and analyzed by FACS (Becton Dickinson).

### Cloning of JAK1

Total RNA of DU145 cells was used as a template for first-strand cDNA synthesis (iSCRIPT, BioRad, cat. #1708890). JAK1 coding sequence was PCR-amplified (Phusion^®^ High-Fidelity DNA Polymerase; NEB, cat. #M0530S), using the following primers: fw 5′CTCGTACGCTTAATTAACGATGCAGTATCTAAATATAAAAGA′3, rev 5′GAGGGGCGGAATTCCGGATCTTATTTTAAAAGTGCTTCAAAT′3. PCR product was inserted into the BamHI site of pHR′-CMV-(ires)-neo vector using the Gibson Assembly method (New Englad Biolabs). Insert was sequenced to ensure the absence of mutations.

### Generation of LNCaP-JAK1 Cells

Lentiviral particles pseudotyped with the G-protein of the Vesicular Stomatitis Virus (VSV-G), harboring the pHR′-CMV-JAK1-(IRES)-neo lentivector, which encodes for the JAK1 and the neomycin resistance genes, were generated and used for infection as previously described (50). Infected cells were selected with 800-µg/mL G418 (Sigma-Aldrich, cat. #108321-42-2). Individual colonies were expanded and the presence and activity of JAK1 were evaluated by immunoblotting.

### Generation of Cells Depleted for STAT1 or STAT3 Expression

The Clustered Regularly Interspaced Short Palindromic Repeats (CRISPR)/CRISPR-associated nuclease (CRISPR/Cas9) system was employed for the knockout of endogenous STAT1 or STAT3 in LNCaP-JAK1 cells. Small guide RNAs (sgRNAs) targeting *STAT1* (GAGGTCATGAAAACGGATGG), *STAT3* (GCAGCTTGACACACGGTACC), or control *GFP* (GGGCGAGGAGCTGTTCACCG) genes were designed using the crispr.mit.edu web tool (52) and cloned into the BsmBI site of pXPR lenti-CRISPR plasmid [encoding for Puromycin resistance (53)]. Lentiviral particles containing the pXPR lentivectors and the above sgRNAs were prepared as described above and used for infection of LNCaP-JAK1 cells. Clones were selected (2-µg/mL puromycin) and the absence of either STAT1 or STAT3 expression was evaluated by immunoblotting.

### Stable Isotope Labeling by Amino-Acid (SILAC) Analysis

LNCaP and LNCaP-JAK1 cells were grown in RPMI devoid of lysine and arginine (Thermo, cat. #A2494401), supplemented with 10% dialyzed FCS (Biological Industries, cat. #04-011-1A) and antibiotics for 10 cell divisions (~3 weeks). “Heavy” culture medium was supplemented with ^13^C_6_^15^N_2_-lysine (146 mg/mL, cat. # CNLM-291-H) and ^13^C_6_^15^N_4_-arginine (84 mg/mL cat. #CNLM-539-H) both from Cambridge Isotope Laboratories. “Light” labeled culture medium was supplemented with unmodified lysine and arginine at the same concentrations. To avoid potential bias in the analysis, in any given condition, cultures were labeled with heavy (H) or light (L) amino acids, or with the reciprocal labeling. Each H–L pair was repeated twice. Thus, quantification of each condition was based on four independent replicates. Labeled cells, either treated or untreated, were trypsinized, counted, and washed twice in cold PBS. Samples were digested by trypsin and analyzed by liquid chromatography tandem-mass spectrometry (LC-MS/MS) on Q Exactive hybrid quadrupole-Orbitrap mass spectrometer (Thermo Scientific). The data were analyzed and quantified with MaxQuant 1.5.2.8 (54), using the human Uniprot database. Proteins were identified with false discovery rate (FDR) < 0.01. Proteins that exhibited differential expression (|log_2_ ratio| ≥ 0.5) in at least three out of four replicates, in any given condition, were chosen for further comparisons. Proteomic analysis of the samples was done at the biomedical core facility at the genomic center (Technion, Israel).

### Statistical Analyses

Data are expressed as means ± SE. Significant differences in mean values were assessed by one-tailed Student’s *t*-test. A value of *p* ≤ 0.05 was considered significant. All experiments were repeated at least three times.

## Results

### JAK1 Expression in LNCaP Cells Restoring IFNα Sensitivity and Altering IL-6 Signaling

**Figure 1:**
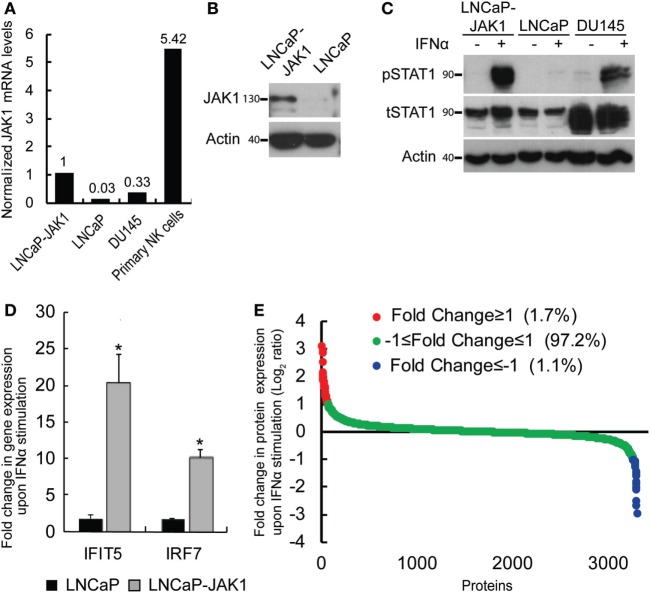
Reactivation of interferon (IFN) signaling by JAK1 expression in LNCaP cells. **(A)** qRT-PCR analysis of JAK1 mRNA levels in different cells. The graph depicts expression levels, relative to GAPDH, in a typical experiment. Levels in LNCaP-JAK1 cells were taken as 1. **(B)** Immunoblot analysis of JAK1 expression in LNCaP-JAK1 and LNCaP cells. Actin served as loading control. **(C)** Immunoblot analysis of pSTAT1 and tSTAT1 in LNCaP-JAK1, LNCaP, and DU145 cells, activated with 200 U/mL of IFNα for 2 h. **(D)** qRT-PCR analysis of fold change in gene expression (normalized to GAPDH expression) of IFIT5 and IRF7 in LNCaP-JAK1 and LNCaP cells, following IFNα stimulation (24 h, 200 U/mL). The average expression level of untreated cells was taken as 1. *p < 0.05. **(E)** Graph depicts average fold change in expression for each protein (*n* = 4) of ~3,800 identified proteins in LNCaP-JAK1 cells, upon treatment with IFNα (200 U/mL, 16 h).

In a previous study, we showed that interferon signaling in LNCaP cells is hampered by biallelic inactivating mutations in the JAK1 kinase, and by epigenetic silencing of JAK1 and of multiple interferon stimulated genes (ISGs), resulting in hypersensitivity to viral infections (15). This study raised the question of the relative contributions of the lack of expression of JAK1 or of ISGs to the susceptibility of these cells to viral oncolysis. To directly address the contribution of JAK1, we transduced LNCaP cells with a lentivector encoding for JAK1. Single clones, resistant to G418, were selected and probed for JAK1 expression. Figure 1A shows the relative expression levels of JAK1 (measured by qRT-PCR) in a representative single clone (LNCaP-JAK1, used here and throughout this study), as compared with the parental LNCaP cells, DU145 interferon-responsive PCa cells and primary natural killer (NK) cells. In accord with both epigenetic silencing and nonsense-mediated decay of the JAK1 message (13–15), in parental LNCaP cells, only very low levels of JAK1 mRNA were detected. In LNCaP-JAK1 cells, JAK1 mRNA levels were comparable with the levels observed in DU145 cells, and lower than the levels observed in the primary NK cells. Based on this, we conclude that ectopic expression of JAK1 in the selected colony falls within a physiological range. Protein expression of JAK1 in LNCaP-JAK1, but not in parental LNCaP cells, was further confirmed by immunoblotting (Figure 1B).

To test for the functionality of the ectopically expressed JAK1, we stimulated LNCaP-JAK1, LNCaP, or DU145 cells with interferon-α (IFNα, 200 U/mL, 2 h), and probed for the levels of total STAT1 (tSTAT1), tyrosine-phosphorylated STAT1 (pSTAT1), and actin (loading control) in extracts of stimulated and unstimulated cells, by immunoblotting. While LNCaP cells were insensitive to IFNα stimuli, LNCaP-JAK1 and DU145 cells showed dramatic increases in pSTAT1 levels upon stimulation (Figure 1C). A slight IFNα-induced increase in tSTAT1 levels was also observed in LNCaP-JAK1 cells. Of note, IFNα also induced phosphorylation of STAT2 (see below). Moreover, qRT-PCR assessment of the levels of expression of known ISGs (shown here IFIT5 and IRF7) showed a marked induction by IFNα in LNCaP-JAK1, but not in LNCaP cells (Figure 1D). To complement these analyses, we performed stable isotope labeling with amino acids in cell culture (SILAC), combined with mass-spectrometry (MS), to measure IFNα-induced proteome changes in LNCaP-JAK1 cells. The graph in Figure 1E depicts the average fold change in protein expression upon IFNα stimulation (expressed as log_2_ ratio; *n* = 4; ~3,800 detected proteins; Table S1 in Supplementary Material; “Detected in any SILAC exps.” Tab). The expression of the vast majority of these proteins (~97%) did not differ between treated and untreated cells (Figure 1E, green dots). Low percentages of proteins were either up or downregulated more than twofold (red or blue dots, respectively, Figure 1E). Gene-ontology (GO) analysis of the subset of upregulated proteins revealed significant enrichment of ISGs (24.4%, *p* < 5E − 10). Taken together, these data show that ectopic expression of JAK1 restores IFNα signaling and ISG upregulation in LNCaP-JAK1 cells.

**Figure 2:**
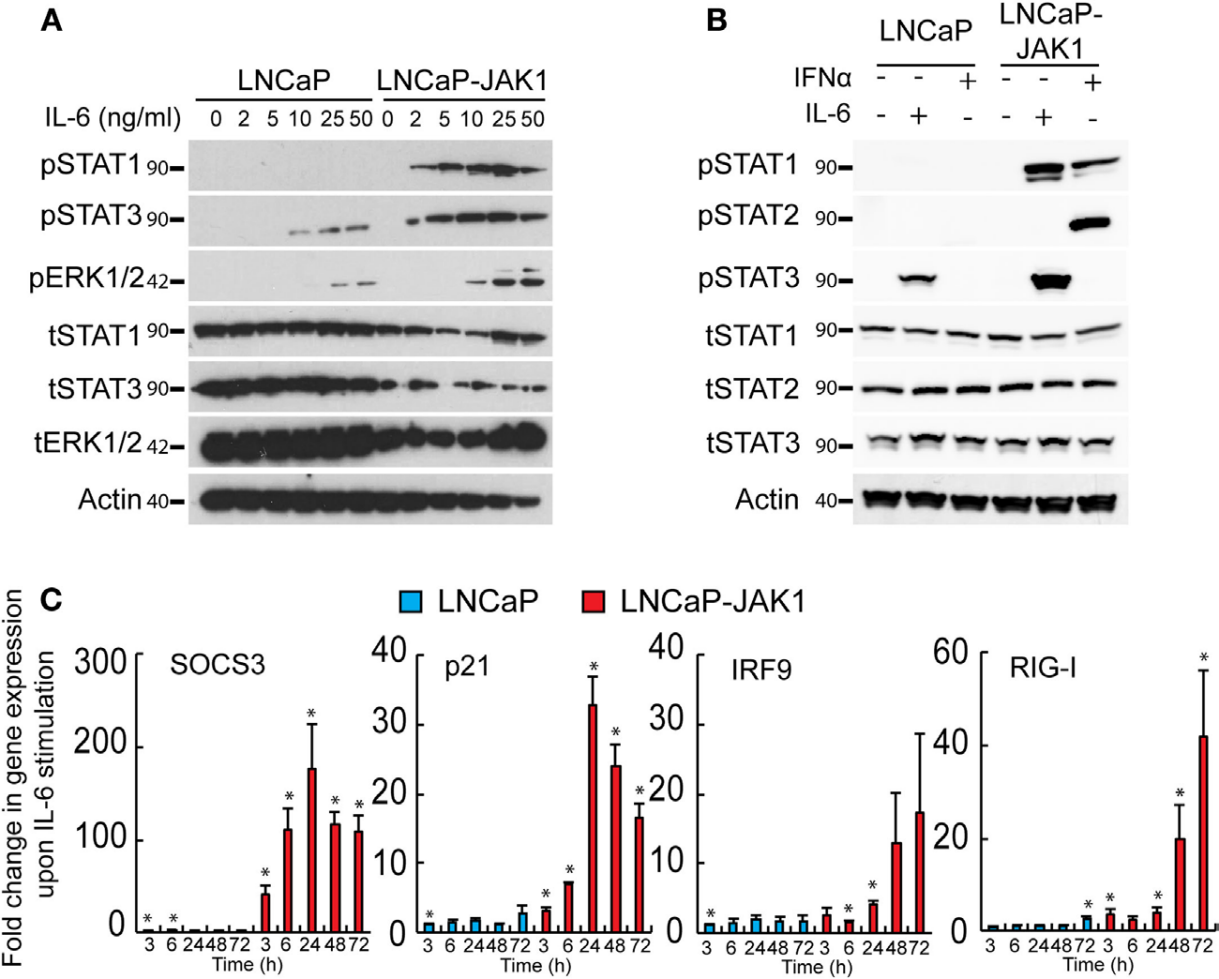
JAK1 expression alters IL-6 signaling in LNCaP-JAK1 cells. **(A)** Immunoblot analysis of STAT1, STAT3, and ERK1/2 phosphorylation. LNCaP and LNCaP-JAK1 cells were exposed to the indicated concentrations of IL-6 for 20 min and processed for immunoblotting. **(B)** Immunoblot analysis of STAT1, STAT2, and STAT3 phosphorylation. LNCaP and LNCaP-JAK1 cells were exposed to IL-6 (5 ng/mL, 20 min) or IFNα (200 U/mL, 20 min) and processed for immunoblotting. **(C)** Transcriptional response of LNCaP and LNCaP-JAK1 to IL-6 stimuli. Cells were incubated with IL-6 (5 ng/mL) for the indicated time periods and mRNA levels of the indicated genes were quantified by qRT-PCR. Graph depicts mean ± SE (*n* = 3) of fold change in mRNA levels upon IL-6 stimuli, normalized to GAPDH expression, in LNCaP or LNCaP-JAK1 cells. *p < 0.05, relative to expression at *t* = 0.

JAK1 transduces signals from multiple cytokine receptors, including those elicited by IL-6 (1, 17, 18). The precise outcome of stimulation of PCa cells with IL-6 is contentious, with studies pointing to either pro- or anti-tumorigenic effects (31, 32, 40, 55–59). LNCaP cells respond to IL-6 and have been employed as a model for IL-6-induced neuroendocrine differentiation (30, 31, 39). To test if JAK1 expression modifies the IL-6 response of LNCaP-JAK1 cells, relative to parental LNCaP cells, we stimulated both cell types with different concentrations of IL-6 (ranging from 0 to 50 ng/mL, Figure 2A). IL-6 (ranging from 10 to 50 ng/mL) resulted in STAT3 phosphorylation (pSTAT3) in LNCaP cells (Figure 2A). LNCaP-JAK1 cells were more sensitive than LNCaP cells to IL-6 stimuli, as higher levels of pSTAT3 were detected; starting at 2 ng/mL and reaching near-maximal levels at 5 ng/mL. The difference in activation levels between these two cell lines was even more pronounced considering the lower levels of tSTAT3 in LNCaP-JAK1. IL-6 may also activate STAT1 and/or the MAPKs ERK1/2 (18). LNCaP cells showed detectable levels of total STAT1 (tSTAT1), but failed to show formation of pSTAT1 at all concentrations of IL-6 tested (Figure 2A). In sharp contrast, LNCaP-JAK1 cells showed pSTAT1 formation already at 2 ng/mL IL-6. Regarding ERK1/2 phosphorylation (pERK1/2), higher levels were observed in LNCaP-JAK1 relative to LNCaP cells (Figure 2A). To further compare and contrast IL-6 and IFNα stimuli in these cellular contexts, we stimulated LNCaP or LNCaP-JAK1 cells with these cytokines and probed for phosphorylation of STAT2 (in addition to STAT1 and 3; Figure 2B). IFNα, but not IL-6, induced robust phosphorylation of STAT2 exclusively in LNCaP-JAK1 cells. Thus, in LNCaP-JAK1 cells, STAT1 was activated by both cytokines, STAT2 by IFNα and STAT3 by IL-6. Accordingly, upon stimulation with IL-6 (5 ng/mL, 3–72 h), the changes in the mRNA levels of known STAT3 and STAT1 target genes (SOCS3, p21, IRF9, and RIG-I) were markedly higher in LNCaP-JAK1, as compared with LNCaP cells (Figure 2C). Taken together, these data show that restoration of JAK1 expression in LNCaP-JAK1 cells alters both quantitative (enhancement of activation of STAT3 and ERK1/2) and qualitative (activation of STAT1) parameters of the IL-6 response, with implications to induction of expression of IL-6 target genes.

### IFNα and IL-6 Signaling Inducing Growth Inhibition and Cell Autonomous Antiviral State in LNCaP-JAK1 Cells

**Figure 3:**
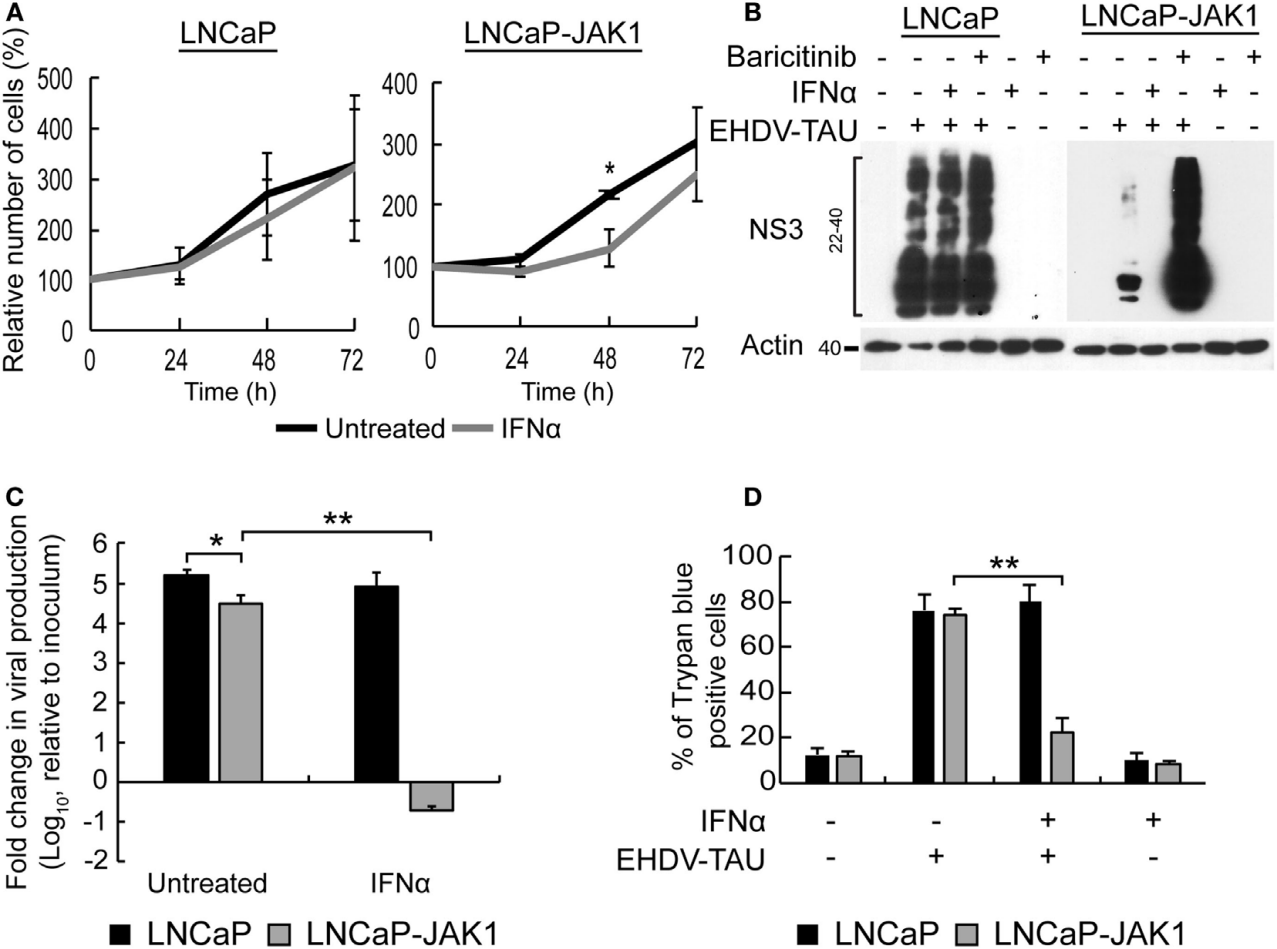
Interferon (IFN)-stimulated antiviral state restricts EHDV-TAU infection in LNCaP-JAK1 cells. **(A)** Cell proliferation analysis by methylene blue assay. LNCaP or LNCaP-JAK1 cells were treated (gray line), or not (black line), with 200 U/mL IFNα, for the indicated time points. Graph depicts the average ± SE relative number of cells (%), amount of cells at *t*_0_ was taken as 100%, *n* = 3. *p ≤ 0.05. **(B)** Immunoblot analysis of NS3 protein. LNCaP or LNCaP-JAK1 cells were treated with either IFNα (200 U/mL, 14 h), or Baricitinib (0.5 µM, 14 h), prior to, and during, infection with EHDV-TAU (moi = 0.5, 48 h). The attenuation of NS3 formation in untreated EHDV-TAU-infected LNCaP-JAK1 cells relative to similarly infected LNCaP cells was variable among different repeats of this experiments (average 57% ± 33% reduction, *n* = 6; shown here an experiment with 53% reduction). **(C)** Fold change in viral titer (relative to inoculum), following EHDV-TAU infection. Indicated cells were treated as in (B) and infected with EHDV-TAU (moi = 0.05, 72 h). Viral titer was measured by plaque assay. *p < 0.05. **p < 0.005. **(D)** Trypan blue exclusion assay. LNCaP or LNCaP-JAK1 cells, treated and infected as in (B), were analyzed by trypan blue exclusion assay to determine percentages of dead cells. Graph depicts mean ± SE (*n* = 5) of the percentage of trypan blue permeable cells with and without IFNα. **p < 0.005.

IFNα is known to inhibit cell proliferation and viral infection. To test the effects of IFNα stimuli and JAK1 expression on these phenomena in LNCaP-based PCa cells, both LNCaP (the parental cell line) and LNCaP-JAK1 cells were treated (or not) with IFNα (200 U/mL) for different time periods. Cell proliferation was measured with a colorimetric assay (Figure 3A) and revealed no significant differences in treated and untreated LNCaP cells. In contrast, IFNα partially inhibited the proliferation of LNCaP-JAK1 cells (significant at 48 h). We next tested the susceptibility of LNCaP and LNCaP-JAK1 cells to viral infection, with or without IFNα treatment (Figure 3B). For this, we employed EHDV-TAU, an adapted orbivirus that efficiently replicates in, and kills LNCaP cells (15). To measure infection, we detected by immunoblotting the levels of the non-structural EHDV-TAU protein 3 (NS3), which is synthesized only in productively infected cells. In accord with our previously published results (15), LNCaP cells were highly susceptible to EHDV-TAU infection, as indicated by the high levels of expression and typical smear-like appearance of NS3, irrespective of IFNα treatment (Figure 3B). In sharp contrast, infection of untreated LNCaP-JAK1 cells resulted in markedly lower NS3 expression (Figure 3B). Baricitinib, a JAK1 inhibitor (60), fully restored the NS3 expression pattern and levels, to those observed in LNCaP cells. In these cells, addition of IFNα abolished NS3 expression, in accord with a role for JAK1 in the inhibition of EHDV-TAU infection in LNCaP-JAK1 cells, (Figure 3B). IFNα also inhibited production of infectious virions only in LNCaP-JAK1 cells (Figure 3C). Moreover, only in IFNα-treated LNCaP-JAK1 cells, protection from EHDV-TAU-induced cell death was observed (Figure 3D). Taken together, restoration of JAK1 expression in LNCaP cells restored IFNα signaling, exposed the cells to IFNα-mediated growth inhibition, and allowed for the cytoprotective, antiviral effects of IFNα, resulting in a block of EHDV-TAU infection.

**Figure 4:**
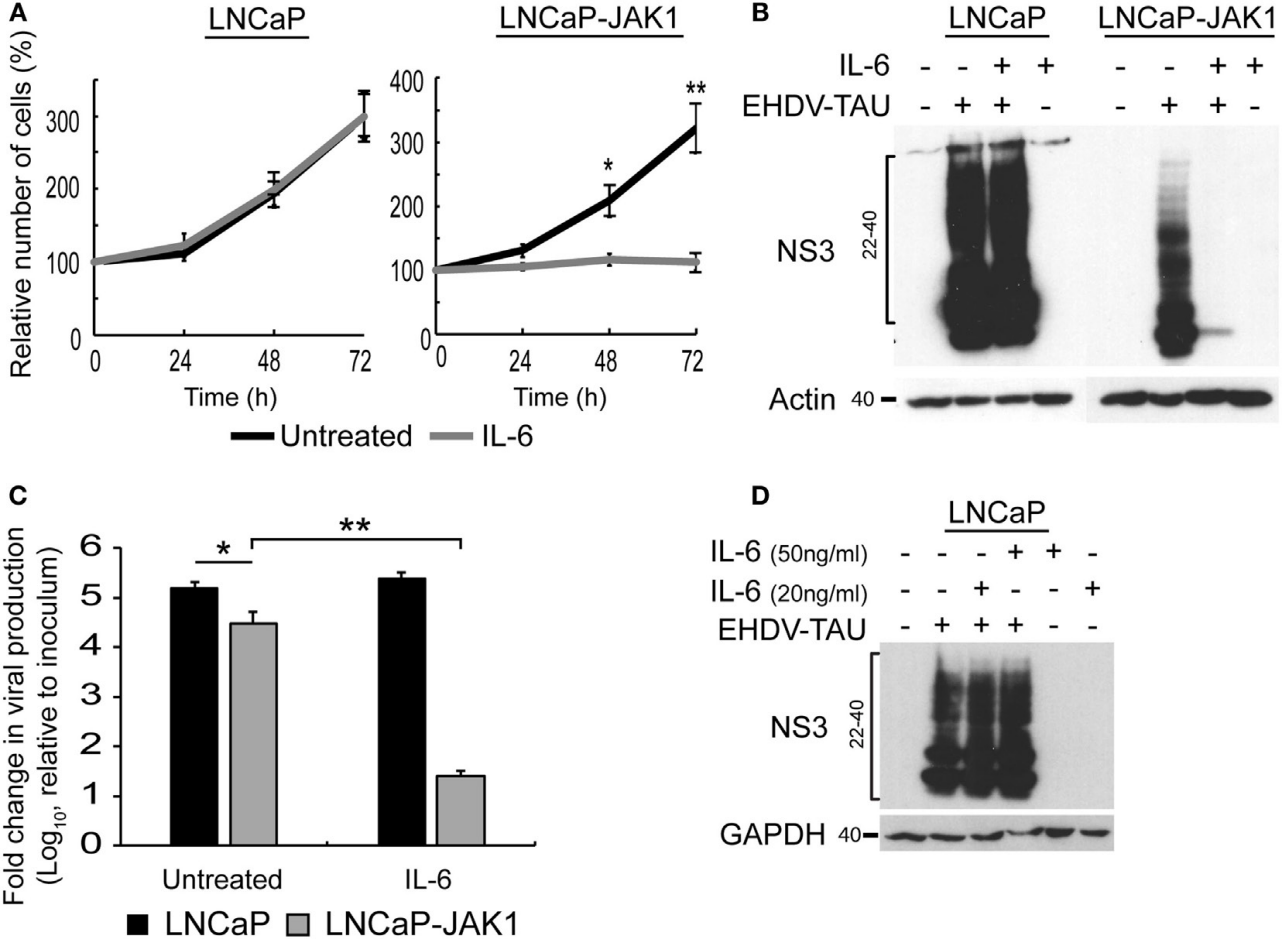
IL-6 signaling in LNCaP-JAK1 inducing proliferation arrest and antiviral state. **(A)** Cell proliferation analysis by methylene blue assay. LNCaP or LNCaP-JAK1 cells were incubated, or not, with IL-6 (5 ng/mL) for the indicated time points. Quantification was in Figure 3A. *p < 0.05. **p < 0.005. **(B)** Immunoblot analysis of NS3 expression upon treatment with IL-6. Indicated cells were treated, or not, with IL-6 (5 ng/mL, 14-h pretreatment and throughout infection), and infected (or not) with EHDV-TAU (moi = 0.5, 48 h). **(C)** Fold change in EHDV-TAU production following IL-6 treatment. LNCaP or LNCaP-JAK1 cells were pretreated (or not) with IL-6 (5 ng/mL, 14 h) and infected with EHDV-TAU in the absence or presence of IL-6. Titers were determined by plaque assay. Significance analysis as in A. **(D)** Immunoblot analysis of LNCaP cells treated, or not, with high concentrations of IL-6 (20 or 50 ng/mL), and infected (or not) with EHDV-TAU (moi = 0.5, 48 h).

We next probed for the functional outcome of IL-6 stimuli on cell proliferation. The growth rates of untreated LNCaP and LNCaP-JAK1 cells were essentially the same (black curves, Figure 4A). IL-6 treatment (5 ng/mL, gray curves, Figure 4A) failed to alter proliferation of parental LNCaP cells, while arresting proliferation of LNCaP-JAK1 cells. Thus, JAK1 mediated anti-proliferative signaling of IL-6; implying that the strong silencing of JAK1 expression in parental LNCaP cells possibly contributed to their thriving in an inflammatory, IL-6-rich tumor micro-environment.

Early studies demonstrated lack of antiviral effect of IL-6 signals, in different cellular models (61). Yet, in LNCaP-JAK1 cells, expression of genes known to mediate innate immunity was upregulated by IL-6 (Figure 2C). To test whether IL-6 exposure affects viral infection, we initially quantified the effect of IL-6 on NS3 levels in EHDV-TAU-infected LNCaP and LNCaP-JAK1 cells. In infected LNCaP cells, high levels of NS3 forms were observed in both treated and untreated cultures. Infection of LNCaP-JAK1 cells in the presence of IL-6 (5 ng/mL), however, resulted in near complete abrogation of NS3 expression, suggesting a strong antiviral effect of this cytokine in JAK1-expressing cells (Figure 4B). Moreover, NS3 expression levels in untreated LNCaP-JAK1 cells were lower than those observed in parental LNCaP cells (Figure 4B), in accord with a possible establishment of antiviral state, *via* autocrine signaling in JAK1-expressing cells. Treatment with IL-6 (5 ng/mL) induced a ~4 orders of magnitude reduction in titer of infectious virions in LNCaP-JAK1 cells, but not in LNCaP cells (Figure 4C), further demonstrating the antiviral effect of this cytokine. To test if higher concentrations of IL-6 reduce EHDV-TAU infection and NS3 expression in parental LNCaP cells, we infected these cells in the presence of IL-6 (20 or 50 ng/mL). No reduction in NS3 expression was observed in any of these conditions (Figure 4D). This is in line with the lack of pSTAT1 formation in LNCaP cells, even at high IL-6 concentrations (Figure 2A). Overall, restoration of JAK1 expression in LNCaP-JAK1 cells results in anti-proliferative and antiviral effects, upon exposure to IL-6.

### IL-6 Signaling Sensitizing LNCaP-JAK1 Cells to Viral Oncolysis

**Figure 5:**
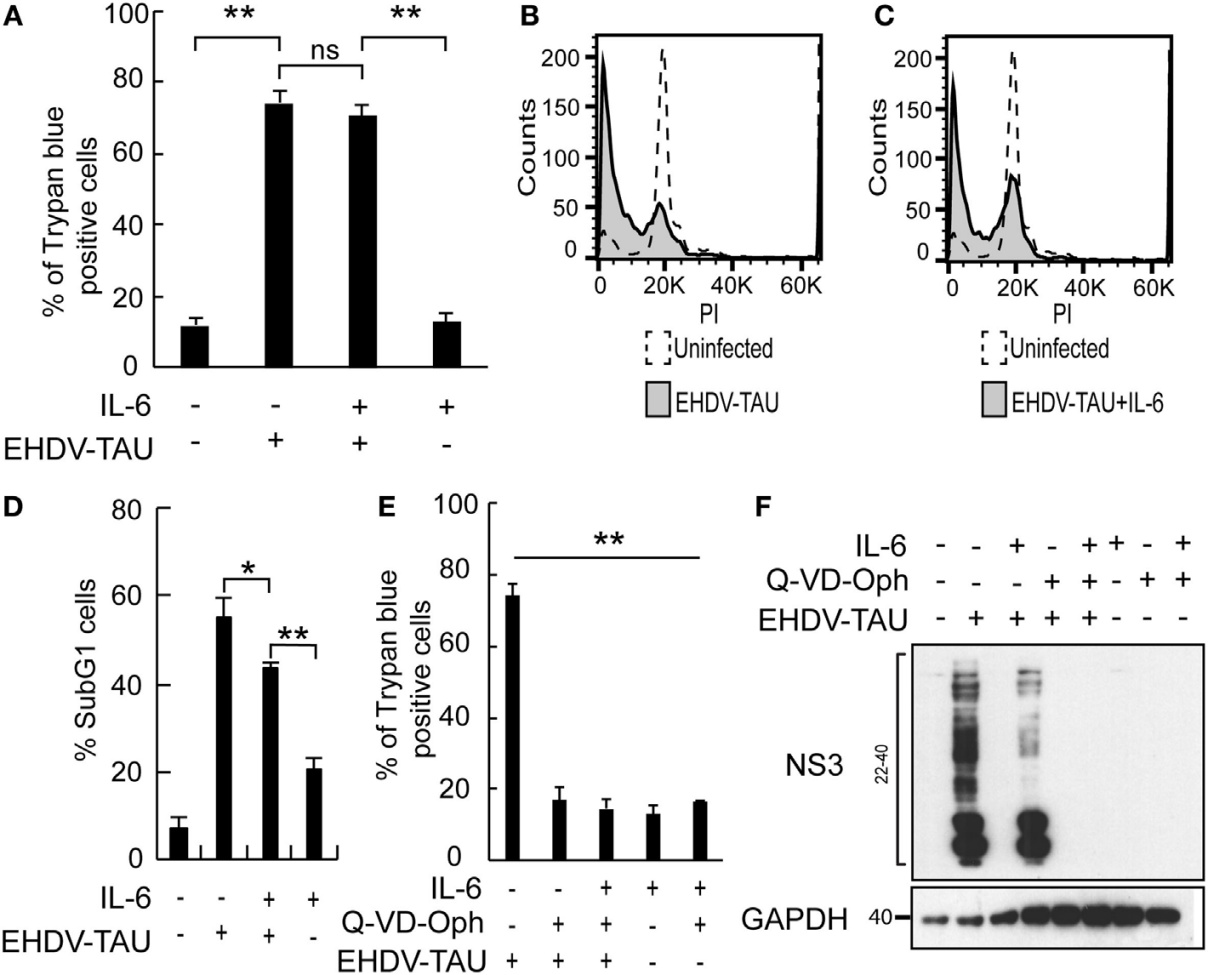
Oncolysis in the absence of productive viral infection in IL-6-treated LNCaP-JAK1 cells. **(A)** Trypan blue exclusion assay of LNCaP-JAK1 cells, treated and/or infected as in Figure 4B. Graph depicts mean ± SE (*n* = 10). **p < 0.005. ns, non-significant. **(B–D)** Fluorescence activated cell sorting analysis to quantify the fraction of sub-G1 cells upon infection and/or IL-6 treatment as in Figure 4B. **(B and C)** Representative graphs of indicated conditions. **(D)** The graph depicts mean ± SD (*n* = 3) *p = 0.04 **p < 0.01. **(E)** Trypan blue exclusion assay of cells treated and/or infected as in Figure 5A, with the exception that Q-VD-OPh was added (throughout pretreatment and infection) to the indicated samples. **(F)** Immunoblot analysis of LNCaP-JAK1 treated, or not, with IL-6 (5 ng/mL) and/or Q-VD-OPh (20 µM), and infected or not with EHDV-TAU (moi = 0.5, 48 h).

**Figure 6:**
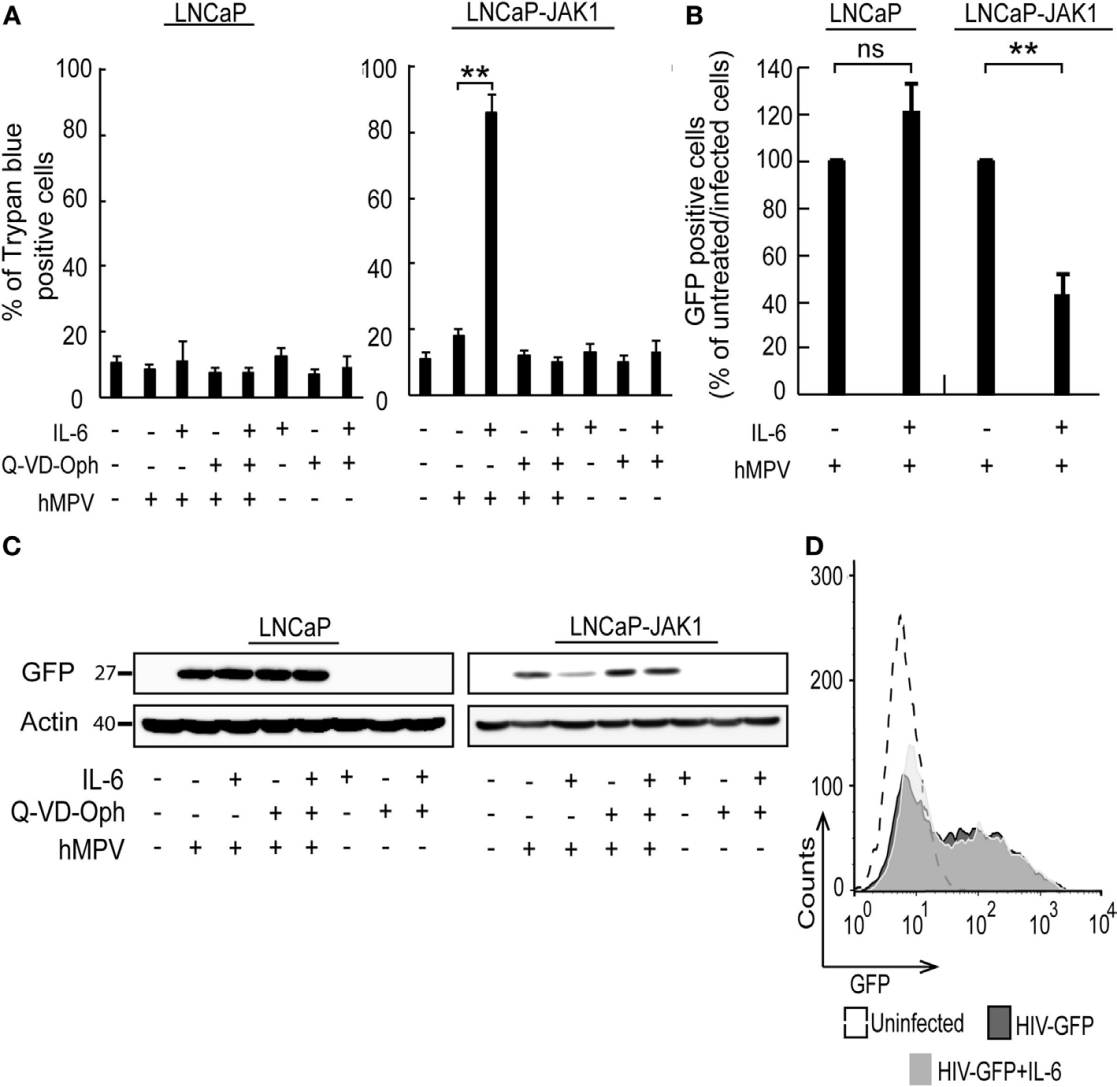
hMPV-GFP infection sensitizing LNCaP-JAK1 to caspase-dependent death in the presence of IL-6. **(A)** Trypan blue exclusion assay of LNCaP and LNCaP-JAK1 cells, treated as in Figure 5E, and infected with hMPV-GFP (“hMPV”; moi = 0.5, 48 h). Graph depicts mean ± SE percentage of dead cells (*n* = 3). **p < 0.005. **(B)** Fluorescence activated cell sorting (FACS) analyses of percentage of infected cells following treatment with IL-6 (5 ng/mL, 14-h pretreatment and throughout infection) and infection with hMPV-GFP (moi = 0.5, 48 h). Bars represent mean ± SE percentage of GFP-positive (infected) cells compared with untreated-infected LNCaP or untreated-infected LNCaP-JAK1 cells. **p < 0.005; ns, non-significant. **(C)** Immunoblot analysis of LNCaP and LNCaP-JAK1 cells treated, or not, with IL-6 (5 ng/mL) and/or Q-VD-OPh (20 µM), and infected or not with hMPV-GFP (moi = 0.5, 48 h). **(D)** FACS analysis of GFP fluorescence levels of LNCaP-JAK1 cells, treated or not with IL-6 (5 ng/mL), and infected with HIV-GFP (48 h).

In spite of the IL-6-induced reductions in productive EHDV-TAU infection of LNCaP-JAK1 cells (Figure 4), visual inspection of these infected cultures suggested massive cell death. To quantify this phenomenon, we employed the trypan blue exclusion assay in each experimental condition (Figure 5A). While untreated and uninfected LNCaP-JAK1 cells showed only minimal loss of membrane impermeability (~10%), infection (48 h) with EHDV-TAU, in the presence or absence of IL-6, resulted in a significant increase in loss of membrane impermeability (~75%). Notably, IL-6 alone had moderate effects on membrane impermeability of LNCaP-JAK1 cells (Figure 5A). To complement these analyses, cells treated and infected as in Figure 5A were stained with PI, and cell cycle was analyzed by FACS. IL-6 treatment induced a slight increase in the percentage of cells exhibiting sub-G1 DNA content (Figure 5D). EHDV-TAU infection induced a marked increase in Sub-G1 fraction (Figure 5B representative curves, Figure 5D average of multiple experiments) similarly to the combination of IL-6 and EHDV-TAU (Figure 5C, representative curves, Figure 5D, average of multiple experiments). Taken together, these results suggest that in both productive or abortive infections, EHDV-TAU is capable of inducing apoptosis in LNCaP-JAK1 cells. To examine if caspases are involved in the death induced by the EHDV-TAU-IL-6 combination, we blocked caspase activity with the pan-caspase inhibitor Q-VD-OPh (62). Caspase inhibition reduced the death of EHDV-TAU-infected LNCaP-JAK1 cells to basal levels, regardless of the presence of IL-6 (Figure 5E). This resembles the effect of Q-VD-OPh on cell viability in infections with the parental EHDV2-IBA (48) or with EHDV-TAU while infecting LNCaP cells (15). To test if inhibition of cell death rescues NS3 production in the presence of IL-6 in LNCaP-JAK1 cells, we probed for NS3 expression upon exposure of these cells to different combinations of IL-6, Q-VD-OPh, and EHDV-TAU (Figure 5F). While rescuing cell viability, Q-VD-OPh failed to rescue NS3 expression in EHDV-TAU-infected, IL-6-treated cells. This shows that concerning EHDV-TAU, the IL-6-induced antiviral effect is not dependent on cell death. As EHDV-TAU kills LNCaP-based cells with high efficiencies (i.e., upon productive infection of untreated cells or upon non-productive infection in IL-6-treated LNCaP-JAK1 cells), we could not address the question if IL-6 sensitizes cells to virally induced cell death. In a previous study (15), we identified replication-competent hMPV, expressing the GFP marker (named “hMPV-GFP”) as an agent that efficiently infects LNCaP cells without causing extensive cell death (within 48 h). Accordingly, we utilized hMPV-GFP to probe the potentiation of virally induced cell death by IL-6. LNCaP or LNCaP-JAK1 cells, treated or not with IL-6 (5 ng/mL, 14 h) were infected or not with hMPV-GFP (moi = 0.5, 48 h). Percentage of cell death was calculated with trypan blue assay. As shown in Figure 6A, the combination of hMPV-GFP and IL-6 resulted in massive cell death only in LNCaP-JAK1 cells, while each of these treatments alone (hMPV-GFP or IL-6) were devoid of cell killing potential. Similarly to EHDV-TAU infection, the caspase inhibitor Q-VD-OPh rescued cell viability of hMPV-GFP-infected, IL-6-treated LNCaP-JAK1 cells, suggesting the involvement of caspases in the induction of cell death (Figure 6A). Next, we probed for IL-6-induced antiviral effects on hMPV-GFP infection. IL-6 treatment of LNCaP-JAK1 cells significantly reduced the percentage of GFP-expressing infected cells, while IL-6 treatment of LNCaP cells was devoid of effects (Figure 6B). Importantly, IL-6 reduced GFP expression, while addition of Q-VD-OPh fully restored GFP expression levels in IL-6-treated LNCaP-JAK1 cells (Figure 6C). Thus, in the case of hMPV-GFP and in contrast to EHDV-TAU, the infection-restricting effect of IL-6 was dependent on cell death. Importantly, these results demonstrate that IL-6 sensitizes JAK1-expressing LNCaP cells to virally induced cell death.

To further test the enhancement of death of infected cells by IL-6, we chose to employ a human immunodeficiency virus (HIV)-based vector, encoding for GFP marker (HIV-GFP), as lenti vectors are designed to infect cells while maintaining cell viability and function. Both IL-6-treated or untreated LNCaP-JAK1 cells displayed similar high levels of GFP expression upon HIV-GFP infection (Figure 6D), implying that IL-6 treatment did not perturb viability and function of infected cells. Taken together, the differential effects of IL-6 on production of virally encoded proteins and cell death demonstrate the specificity of the antiviral effects of IL-6 toward different viral infection programs.

### JAK1-Related Changes to Proteome of PCa Cells

**Figure 7:**
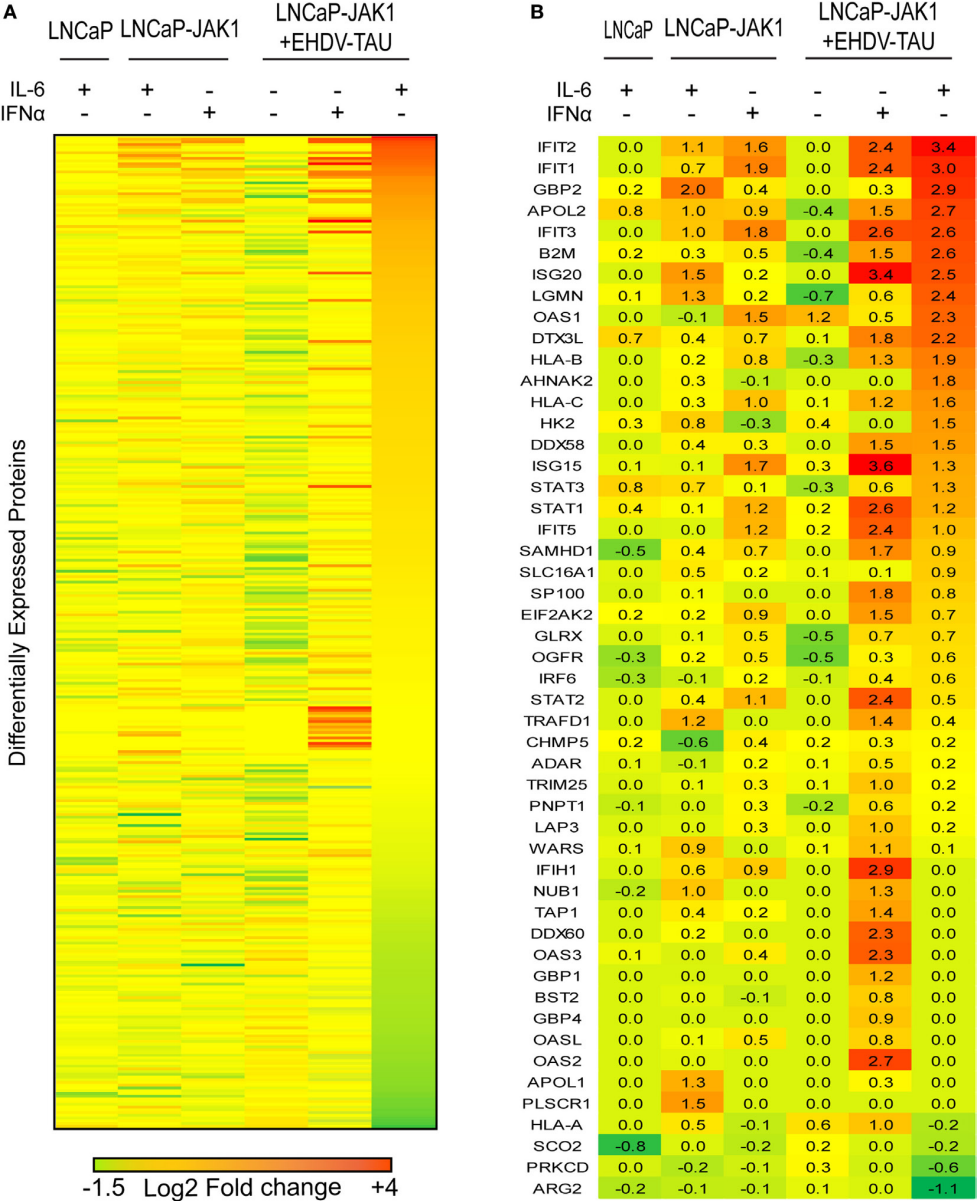
EHDV-TAU infection dramatically changing the proteome of IL-6-treated LNCaP-JAK1 cells. **(A)** Heatmap of differently expressed proteins (|log_2_ ratio| of average (*n* = 4) ≥ 0.5) in LNCaP or LNCaP-JAK1 cells under different conditions. Labeled cells were treated with IFNα (200 U/mL) or IL-6 (5 ng/mL) for 16 h, followed by EHDV-TAU infection (moi = 0.5, 24 h, in the presence of IL-6 or IFNα) where indicated. Sorting of the 363 differently expressed proteins––high (red) to low (green)––was according to the most extensively altered condition (EHDV-TAU + IL-6). **(B)** Heatmap of 50 differently expressed ISGs in each of the indicated conditions [generated by crossing the list of 363 differently expressed proteins with a list of 500 known ISGs (15, 63), sorted as in **(A)**]. Average (*n* = 4) |log_2_ ratio| fold change in expression is presented.

To quantify changes in protein expression of LNCaP-based cells upon re-expression of JAK1, cytokine treatment and/or infection, we applied SILAC to cells stimulated with IFNα or IL-6 and infected or not with EHDV-TAU. In repeated experiments (*n* = 4), ~3,800 proteins were identified by LC-MS/MS (listed in Table S1 in Supplementary Material; “Detected in any SILAC exps.” tab), of which 2,658 were shared by all experiments (listed in Table S1 in Supplementary Material; “Shared by all SILAC exps.” tab). Of these, 363 exhibited differential expression (|log_2_ ratio| ≥ 0.5) in at least three out of four replicates in any given condition. These proteins were chosen for further analysis (see Materials and Methods, average values for each protein in each treatment are shown in Table S1 in Supplementary Material; “Differential expression” tab). The comprehensive differential expression of these 363 proteins (|log_2_| ratio of change in expression in treated versus untreated cells of the same type) is depicted as a heatmap in Figure 7A. Summing of changes in expression for all proteins in each treatment revealed that the most extensive changes occurred for LNCaP-JAK1 cells, treated with IL-6 and infected with EHDV-TAU. Accordingly, the entire heatmap was ordered relative to the highest-to-lowest fold change in this condition.

Due to the crucial role of ISGs in regulating cellular antiviral state, we centered our analyses on the 50 ISGs products that were identified in the above 363 proteins (Figure 7B). To estimate the contribution of JAK1 to IL-6-mediated changes in ISG expression, we compared LNCaP to LNCaP-JAK1 cells, both treated or not with IL-6 (5 ng/mL; 16 h). Upon IL-6 treatment, only three ISGs were upregulated in LNCaP cells, as opposed to 16 in LNCaP-JAK1 cells (Figure 7B). This is in accord with IL-6-mediated phosphorylation of STAT1 (Figure 2A) and inhibition of viral infection in LNCaP-JAK1, but not LNCaP, cells (Figure 4B). As both IL-6 and IFNα induced antiviral states in LNCaP-JAK1 cells, we compared the profiles of upregulated ISGs in each condition. Here, IFNα treatment of LNCaP-JAK1 cells induced 17 out of the 50 ISGs. Notably, comparison of these ISGs to the 16 ISGs induced by IL-6 revealed that only five (IFIT1-3, APOL2, IFIH1) were common to both conditions. Thus, the two cytokines induce different, but partially overlapping sets of ISGs, correlating with the different sets of STATs activated by these cytokines.

Viral infection modifies the cellular response to cytokine signaling (e.g., through activation of pattern recognition receptors). Next, we addressed the induction of ISGs by EHDV-TAU infection in the absence or presence of IL-6 or IFNα. EHDV-TAU infection of untreated LNCaP-JAK1 cells induced only two ISGs (OAS1 and HLA-A). This minimal induction, which apparently failed to block productive infection in these cells, was potentiated by both IL-6 or IFNα, as 27 or 37 ISGs were induced by the combination of EHDV-TAU infection and these cytokines, respectively (Figure 7B). Analysis of these two sets of ISGs revealed that the majority (20) of ISGs were shared, in accord with the induction of antiviral state by both cytokines. The 17 ISGs unique to the IFNα + EHDV-TAU condition (IFIH1, TRAFD1, WARS, NUB1, TRIM25, LAP3, TAP1, DDX60, OAS3, GBP1, BST2, GBP4, OASL, ADAR, PNPT1, OAS2) may contribute to the cytoprotective antiviral-state, induced by IFNα.

Analysis of the 363 proteins with significant changes in expression (in any condition) revealed 71 proteins showing reduced expression (log_2_ ratio ≤ −0.5) in the EHDV-TAU + IL-6 combination. GO annotation (statistical overrepresentation test) (64) of this subset revealed overrepresentation for proteins involved in “DNA metabolic processes” (*p* < 10^−8^), “DNA replication” (*p* < 10^−6^), and “metabolic processes” (*p* < 10^−5^) (Figure S1 in Supplementary Material). Remarkably, only seven of these 71 proteins were downregulated in the IFNα + EHDV-TAU condition. GO annotation analysis of these seven proteins (PFDN2, CAD, MCM6, MCM7, STMN1, RRM1, CKS1B) failed to reveal statistically significant overrepresentation for any process. Taken together, these results demonstrate the induction (in JAK1-expressing cells) of ISGs by cytokines (IL-6 and IFNα) and the augmentation of this induction by viral infection. In Figure 2, we observed the induction of SOCS3 mRNA by IL-6 in LNCaP-JAK1 cells. SOCS3 is a negative regulator of IL-6 (65). To test if SOCS3 protein is induced by IL-6 (with or without infection), we pretreated (or not) LNCaP-JAK1 cells with IL-6 (5 ng/mL, 12 h) and subsequently infected or not these cells with EHDV-TAU (moi = 0.5, 48 h). Immunoblot of pSTAT1 in these conditions revealed pSTAT1 formation, at this late time point, in “IL-6” and “IL-6 + EHDV-TAU” conditions. Notably, no increase in SOCS3 was observed in any of the conditions examined, relative to uninfected/untreated cells. Taken together, these data imply that SOCS3 is regulated posttranscriptionally in LNCaP-JAK1 cells, and that absence of its induction at protein level supports prolonged STAT1 signals (Figure S2 in Supplementary Material). The differences between cytoprotection of infected cells in the presence of IFNα, as opposed to viral-mediated oncolysis in the presence of IL-6 may stem from the down regulation of regulators of cellular metabolism by IL-6 and/or the upregulation of additional ISGs set by IFNα.

### Differential Contribution of STATs to the Antiviral State and Growth Inhibition by IL-6

**Figure 8:**
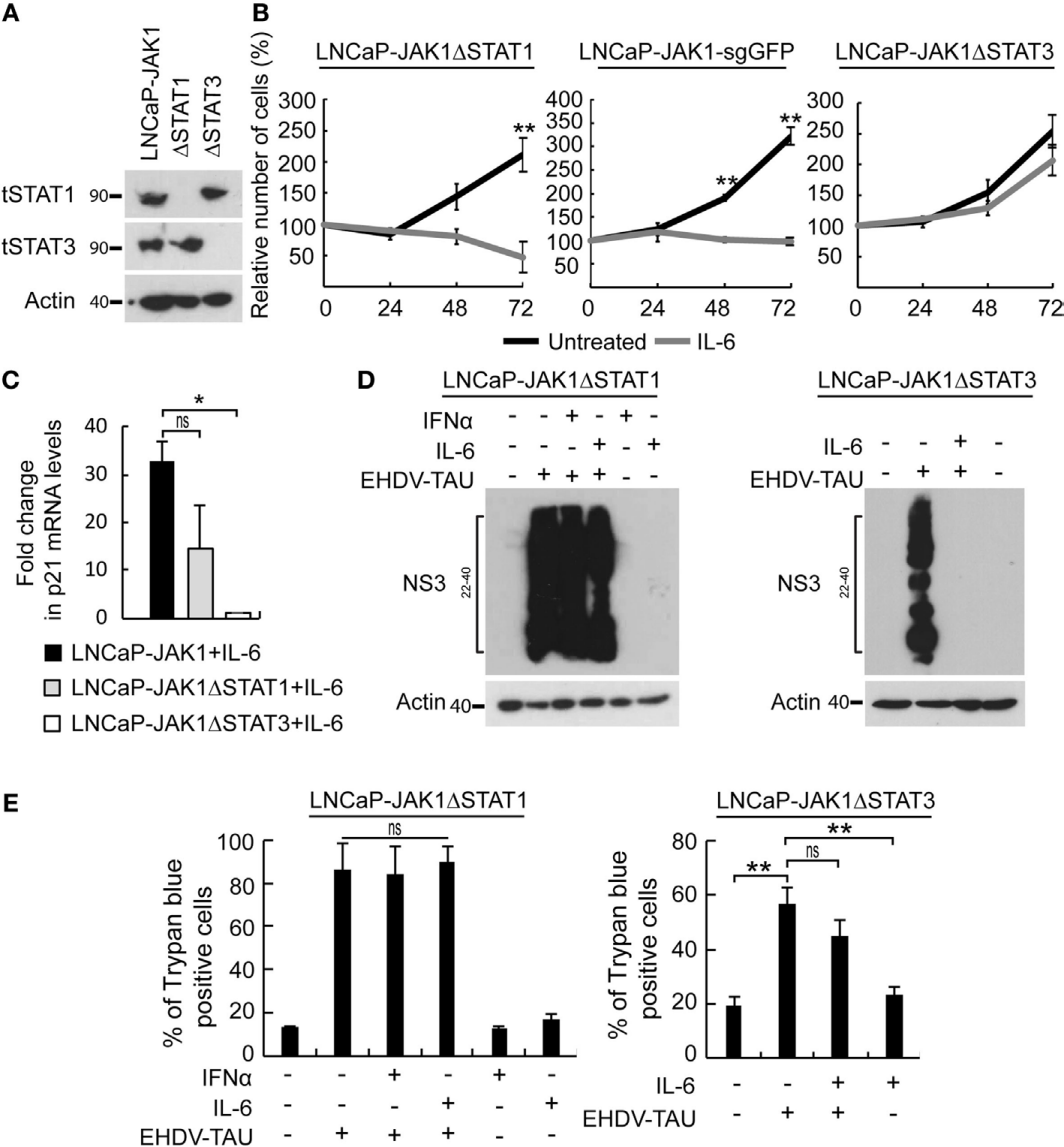
Differential contribution of STAT1 and STAT3 to IL-6-induced phenomena. **(A)** Verification of STAT expression in CRISPR-Cas9-mediated knockout clones of STAT1 or STAT3. LNCaP-JAK1, LNCaP-JAK1ΔSTAT1 (ΔSTAT1), and LNCaP-JAK1ΔSTAT3 (ΔSTAT3) cells were probed for expression of STAT1 (tSTAT1) and STAT3 (tSTAT3) by immunoblotting. **(B)** Cell proliferation assay (methylene blue). Indicated cells were treated (gray curves) or not (black curves) with IL-6 (5 ng/mL) for the indicated time periods. **p < 0.005. **(C)** Changes in p21 expression. qPCR analyses of p21 mRNA levels were performed with indicated cells, treated or not with IL-6 (5 ng/mL, 24 h). Graph depicts mean ± SE (*n* = 3) of fold changes in p21 expression (normalized to GAPDH expression). *p < 0.05. **(D)** Immunoblot analysis of EHDV-TAU infection. Extracts of the indicated cells, treated or not, with IFNα (200 U/mL, 14 h) or IL-6 (5 ng/mL, 14 h); and infected, or not, with EHDV-TAU (moi = 0.5, 48 h), were immunoblotted for NS3 expression. **(E)** LNCaP-JAK1ΔSTAT1 and LNCaP-JAK1ΔSTAT3 cells were treated and infected as in **(D)**. Cells were analyzed by trypan blue exclusion assay to determine percentages of dead cells. Graphs depict mean ± SE of the percentage of dead cells (*n* = 2, LNCaP-JAK1ΔSTAT1; *n* = 4, LNCaP-JAK1ΔSTAT3; **p < 0.005; ns, non-significant).

In the above-described experiments, IL-6 activated STAT1 and STAT3 and induced three main phenomena in LNCaP-JAK1 cells: antiviral state, cytostasis, and oncolysis upon non-productive EHDV-TAU infection. To dissect the relative contribution of each of these STAT proteins to these processes, we used CRISPR-Cas9 system to knock-out STAT1 or STAT3 in LNCaP-JAK1 cells, obtaining either LNCaP-JAK1ΔSTAT1 or LNCaP-JAK1ΔSTAT3 clones, respectively (Figure 8A). Stimulation of these clones with IL-6 resulted in the phosphorylation of the retained STAT protein (data not shown). The proliferation of LNCaP-JAK1ΔSTAT1 and control LNCaP-JAK1 cells expressing sgRNA against GFP (LNCaP-JAK1-sgGFP cells) was significantly inhibited by IL-6. In contrast, IL-6 failed to significantly inhibit proliferation of LNCaP-JAK1ΔSTAT3 cells (Figure 8B). In line with the IL-6-mediated growth inhibition, only LNCaP-JAK1 and LNCaP-JAK1ΔSTAT1 cells exhibited upregulation of p21 (a cyclin-dependent kinase inhibitor) expression upon stimulation with IL-6 (Figure 8C). To probe for STAT1 or STAT3 roles in IL-6-mediated inhibition of infection, we tested the effect of IL-6 (5 ng/mL, 14 h) on the knockout cells with or without infection with EHDV-TAU (48 h, multiplicity of infection moi = 0.5). In infected LNCaP-JAK1ΔSTAT1, high levels of NS3 expression were obtained regardless of IL-6 treatment (Figure 8D). In contrast, IL-6 induced a potent antiviral state in LNCaP-JAK1ΔSTAT3 cells (Figure 8D). This antiviral effect correlated with an increase in DDX58/RIG-I mRNA expression, which was of similar magnitude to that observed in LNCaP-JAK1 cells under the same conditions (~fourfold induction, measured by qRT-PCR). As IFNα mediates antiviral state through STAT1, we also probed for the ability of this cytokine to block EHDV-TAU infection in LNCaP-JAK1ΔSTAT1 cells. As expected, IFNα failed to block this infection (Figure 8D). Not surprisingly, the massive expression of NS3 in LNCaP-JAK1ΔSTAT1 (irrespective of cytokine treatment, Figure 8D) was accompanied by high levels of EHDV-TAU-induced cell death (Figure 8E). EHDV-TAU-induced oncolysis was also observed in LNCaP-JAK1ΔSTAT3 cells in either absence or presence of IL-6 (Figure 8E), resulting in productive or non-productive infection, respectively (Figure 8D). Further support for the prominent role of STAT1 activation in the restriction of EHDV-TAU infection in LNCaP-JAK1 cells and in EHDV-TAU induced oncolysis by non-productive infection comes from the observed effects of IFNγ, known to mediate antiviral effects through STAT1 homodimer formation (66). Indeed, in LNCaP-JAK1 cells, IFNγ activated STAT1 but not STAT3 (Figure S3A in Supplementary Material), abrogated NS3 production in EHDV-TAU-infected cells (Figure S3B in Supplementary Material), and supported oncolysis by non-productive viral infection (Figure S3C in Supplementary Material). SILAC analyses of IFNγ-treated LNCaP-JAK1 cells revealed extensive ISGs induction (35 of the above 50 identified ISGs), demonstrating the ability of STAT1 homodimers to efficiently mediate activation of ISGs expression (Figure S3D in Supplementary Material). Taken together, these experiments demonstrate different functions for STAT1 and STAT3: STAT1 is necessary for the cell autonomous antiviral effect of IL-6, while STAT3 mediates IL-6-induced anti-proliferative effect. STAT3 also partially contributed to EHDV-TAU-induced oncolysis in the presence of IL-6, which occurs in the absence of productive infection.

### Analysis of Advanced PCa Patient Samples Revealing a Network of Gene Expression Related to JAK1

An early study that analyzed 12 cell cultures derived from PCa showed that IL-6 expression was upregulated, and that the GOs “JAK–STAT cascade” and “response to virus” were overrepresented in advanced-stage PCa samples (67). In addition, IL-6 and STAT3 were shown to promote a PCa stem-like cell phenotype, which is associated with enhanced metastatic potential (68). As EHDV-TAU successfully kills IL-6-treated LNCaP-JAK1 cells, which express JAK1 and STAT1/3 target genes, we interrogated [with cBioPortal (69)] gene expression in patient-derived metastatic PCa samples (70). This study comprised 81 individuals, including 51 with clinical histological features of castration-resistant prostate adenocarcinoma, and 30 with features of castration-resistant neuroendocrine PCa. JAK1 gene amplification was found in 18.2% of the neuroendocrine samples, and in 6.3% of the adenocarcinoma samples. Impressively, when considering both copy number amplification and mRNA upregulation, 53% of the samples showed increased JAK1 expression. Figure S4 in Supplementary Material shows the network [as presented by cBioPortal (69)] formed by genes jointly upregulated with JAK1 in at least 50% of the samples. The vast majority of these genes are transcriptional targets of STATs (including DDX58/RIG-I, IFNAR2, TRIM25, SOCS3, and HLA isoforms; Figure S5 in Supplementary Material). Taken together, these data suggest that metastatic progression, which can be triggered by IL-6 (22, 24), may alter the susceptibility of PCa cells to viral infection and virally induced-cell death, by changing expression of ISGs.

## Discussion

The main results of our study show that in PCa cells expressing functional JAK1, IL-6 restricts the infection of specific viruses *via* induction of a cell autonomous antiviral state and/or sensitization to cell death upon infection. Moreover, these IL-6-induced phenomena are in contrast with the response elicited by IFNα, as the latter induces an antiviral state that is cytoprotective. The differential outcomes resulting from activation with different cytokines and viral infections are summarized in Table 1.

**Table 1:** The phenotypic outcomes and STAT activation.

	Phenotypic outcome				Activation		
	Cytostasis	Viral-mediated oncolysis	Cytoprotection	Restriction of infection	STAT1	STAT2	STAT3
IL-6	+	+	–	EHDV-TAU+ hMPV-GFP+ HIV-GFP−	+	−	+

IFNα	+/−	−	+	EHDV-TAU+ hMPV-GFP ± NS	+	+	−

IFNγ	NT	+	–	EHDV-TAU+	+	−	−

*Results refer to LNCaP-JAK1 cells*.

*“+” indicates occurrence and “−” indicates absence*.

*NT, not tested; NS, data not shown*.

Table 1 summarizes the following findings: (i) STAT2 activation correlated with cytoprotection, (ii) STAT1 activation was necessary for antiviral state, and (iii) STAT3 activation mediated IL-6-induced cell growth arrest. As we employed cells with similar genetic background but differing in JAK1 expression (LNCaP versus LNCaP-JAK1), we could identify the roles of JAK1-mediated activation of STAT1 or STAT3 in the induction of antiviral state or cytostasis; as neither cytostasis nor antiviral state occurred in LNCaP cells. Moreover, CRISPR-mediated knockout that eliminated expression of STAT1 or STAT3 in LNCaP-JAK1 cells also abrogated antiviral and cytostasis effects, respectively, supporting the notion that these signal transducers are involved in different cellular programs. Of note even though STAT3 is generally considered as an oncogene (71), recent studies have shown that in PCa cells endowed with wild-type p53 and lacking the PTEN phosphatase [such as LNCaP cells (72, 73)] STAT3 activation correlates with onco-suppressor activities (40). Concerning STAT2, it should be noted that this protein is an essential component of the ISGF3 transcriptional complex, suggesting that only in IFNα-treated cells (the sole condition where we observe pSTAT2) such complex would be active, altering transcription in a manner resulting in cytoprotection. This is in contrast to IL-6-treated cells where cytokine effects are expected to be mediated by STATs 1 and 3. Recently, STAT2 has been described as a negative regulator of STAT1 activity, suggesting that it may have additional regulatory roles which can be modulated by its activation (74). In the present study, we concentrated on activation (phosphorylation) of STATs as this modification is central to their transcriptional activity. However, it should be noted that both JAKs and the unphosphorylated forms of STAT proteins and have been proposed to exert additional regulatory roles through diverse mechanisms. These include protein–protein interactions, localization to distinct intracellular compartments (e.g., mitochondrial localization of STAT3 or nuclear localization of JAKs), epigenetics, and chromatin organization (74–77). The putative contribution of these alternative mechanisms to the biology of the different LNCaP-based cells generated in the present study will be addressed in the future.

Table 1 also shows that EHDV-TAU or hMPV-GFP viruses differently respond to the antiviral programs induced by IL-6 or IFNα. EHDV-TAU infection was strongly restricted by either IL-6 or IFNα, as evident by the strong reduction in both NS3 expression and production of infectious virions. hMPV-GFP infection (as measured by GFP expression) was also inhibited by IL-6. However, and in contrast to NS3 expression, the inhibitory effect of IL-6 on GFP expression, which occurred within the context of massive cell death, could be rescued by caspase inhibition (with Q-VD-OPh). This indicates that the IL-6-induced restriction to hMPV infection can be attributed to caspase-mediated cell death, while this cytokine induces a cell state which is intrinsically refractory to EHDV-TAU infection, independently of the induction of cell death. Moreover, these two viruses also differed in their sensitivity to IFNα, which strongly inhibited EHDV-TAU while having only partial effect on hMPV-GFP (data not shown). These differences are likely rooted in the natural specificity of hMPV toward human cells (78), in contrast to the specificity of EHDV to non-human (ruminants and insects) cells (79). Furthermore, the adaptation process that generated EHDV-TAU (on the basis of the naturally occurring EHDV2-IBA) was carried out in LNCaP cells, which lack functional JAK1 and are thus unable to mount effective antiviral responses (15). Thus, this adaptation process is not predicted to endow the adapted virus with abilities to confront the interferon-based innate immune restrictions specific to human cells. We speculate that the parental viruses of hMPV-GFP and HIV-GFP (that also was not restricted by IL-6), evolved to combat the cell autonomous antiviral responses of human cells (80). The employment of hMPV-GFP also allowed us to specifically address the question if IL-6 sensitizes JAK1-expressing LNCaP cells to virally induced death. In contrast to the lack of induction of cell death by hMPV-GFP alone, exposure of LNCaP-JAK1 cells to IL-6 dramatically potentiated killing by the virus (~90% cell death). These results further underscore the importance of taking into consideration the composition of the microenvironment while selecting virotherapy agents, as the outcome of the interaction of viruses with cancer cells can be dramatically altered by cytokine signaling. Importantly, in order to be clinically employable, oncolytic viruses need to discriminate between tumor and non-tumor cells. The fact that EHDV-TAU infection is blocked in non-tumorigenic cells (15), yet, efficiently kills either tumor cells defective in JAK1 signaling (killing that is accompanied by productive infection) or JAK1-positive tumor cells stimulated with cytokines such as IL-6 (killing that is accompanied by non-productive infection), suggests that EHDV-TAU may serve as an efficient oncolytic agent for specific human malignancies.

Stable isotope labeling by amino-acid analyses revealed three main changes in the proteome of treated cells: (i) IFNα and IL-6 induced partially overlapping sets of ISGs. This partiality in overlap, while possibly indicating a core of antiviral genes with the potential of blocking infection of viruses such as EHDV-TAU (e.g., IFIT1-3, APOL2, IFIH1), likely contributes to the differences in their antiviral programs (e.g., their differential potency in restricting hMPV-GFP). (ii) The combination of viral infection and cytokine stimulation results in much more extensive changes to the proteome, compared with either treatment alone. Different molecular mechanisms have been proposed to explain how infection can induce heterologous cellular responses to antiviral cytokine signaling. These include, differential posttranslational modifications of the cytokine receptors or JAK–STAT signal mediators, the induction (or activation) of transcription factors that cooperate with STATs (e.g., IRF3 and/or IRF7) to induce ISG expression or modulate chromatin states at target gene loci, or the modification of factors that mediate translation, which may also profoundly alter patterns of protein expression (81). (iii) The differences between the proteome changes, induced by the combination of EHDV-TAU with IFNα, or with IL-6, were apparent by the more extensive induction of ISGs in the EHDV-TAU + IFNα condition; and by the downregulation of a set of proteins related to cell and DNA metabolism in the EHDV-TAU + IL-6 condition. We speculate that these differences are at the basis of the cytoprotective nature of the IFNα response as opposed to oncolysis in the absence of productive infection, induced by IL-6. Interestingly, the inhibitory potential of STAT signaling on metabolism (e.g., mitochondrial biogenesis and function) was recently proposed as a component of the cellular response to pathogen associated molecular patterns (82).

Oncolytic virotherapy aims at the eradication of tumors through the selective infection and killing of cancer cells and the elicitation of anti-tumor immunity (83, 84). Tumor-induced alterations to multiple molecular features of the cell autonomous antiviral response, including defects in JAK–STAT signaling, expose cancerous cells (e.g., PCa cells) to viral infection and viral-induced cell death (15). A possible origin of such defects is immune-editing of the tumor, a process in which modified tumor cells, which are able to evade immune detection and the anti-proliferative nature of a subset of inflammatory cytokines, are selected (85). Interestingly, acquired defects to IFN-induced JAK–STAT signaling have been proposed as a determinant of resistance to immunotherapy (86, 87). The abilities of oncolytic viruses to exploit such defects, and stimulate anti-tumor immunity, reinforce their potential for the development of novel and effective combination therapy strategies.

## Author Contributions

OD, TP, EB, and ME conceived the experiments. OD carried out the experiments. OD, TP, EB, and ME analyzed the data and wrote the manuscript.

## Conflict of Interest Statement

The authors declare that the research was conducted in the absence of any commercial or financial relationships that could be construed as a potential conflict of interest.
