# Supplementary material for: Interleukin-6 and Interferon-α Signaling via JAK1–STAT Differentially Regulate Oncolytic versus Cytoprotective Antiviral States
Source: Front Immunol. 2018 Jan 30;9:94. doi: 10.3389/fimmu.2018.00094 (PMC5797546; doi:10.3389/fimmu.2018.00094)
Supplement: Supplementary file 2 [file Presentation_1.PDF]

Supplementary Material

Interleukin-6 and Interferon-α Signaling via JAK1-STAT Differentially Regulate Oncolytic versus Cytoprotective Antiviral States

Oded Danziger[1], Tal Pupko[1], Eran Bacharach[1$], Marcelo Ehrlich[1$]

Correspondence: Marcelo Ehrlich, marceloe@post.tau.ac.il, Eran Bacharach, eranba@tauex.tau.ac.il

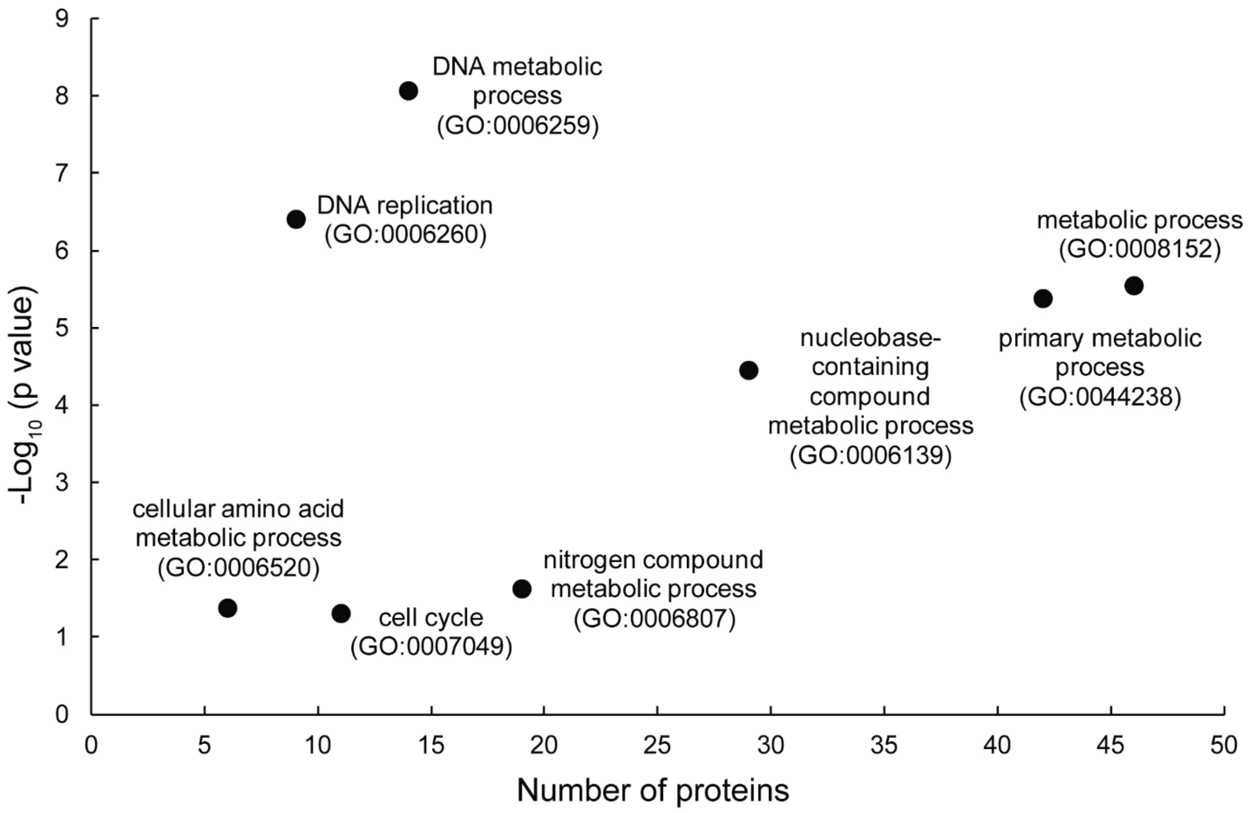

Supplementary Figure 1. EHDV-TAU infection in the presence of IL-6 downregulates expression of proteins involved in DNA replication and associated processes. Graph depicts the eight most significantly enriched cellular processes (GO terms), which were downregulated in IL-6-treated, EHDV-TAU-infected LNCaP-JAK1 cells, relative to naive LNCaP-JAK1 cells. Experimental conditions and protein expression data, obtained by SILAC, were as described in Fig. 7A. X axis: the number of proteins showing downregulated expression in each GO term; Y axis: the p values of the over-representation. GO processes are presented adjacent to numerical identifier.

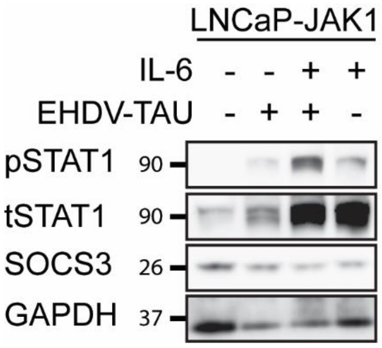

Supplementary Figure S2. Lack of SOCS3 protein induction by IL-6 in LNCaP-JAK1 cells. LNCaP-JAK1 cells were pre-treated (or not) with IL-6 (5 ng/ml, 12 h) and subsequently infected or not with EHDV-TAU (moi=0.5, 48 h). Cell lysates were immunoblotted against the indicated proteins.

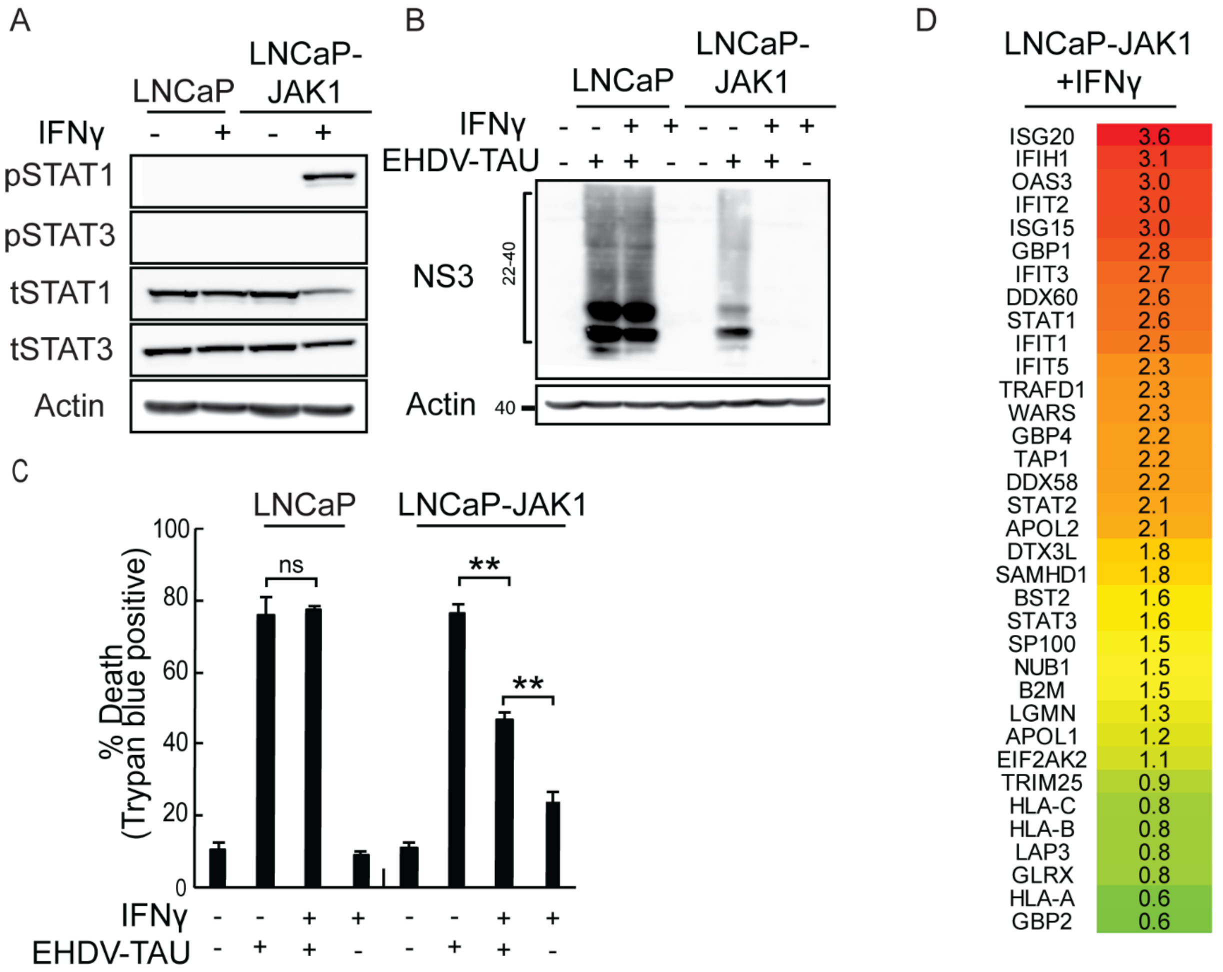

Supplementary Figure 3. Treatment with IFNγ induces STAT1 phosphorylation and partially rescues LNCaP-JAK1 from EHDV-TAU mediated death. (A) Immunoblot analysis of STAT1 and STAT3 phosphorylation. LNCaP and LNCaP-JAK1 cells were exposed to 25 ng/ml of IFNγ for 20 min. **(B)** Immunoblot analysis of IFNγ effect on EHDV-TAU infection. LNCaP or LNCaP-JAK1 cells treated or not with 25 ng/ml of IFNγ (14 h pretreatment and throughout infection), and infected or not with EHDV-TAU (moi=0.5, 48 h). **(C)**. Trypan blue exclusion assay. Indicated cells were treated and infected as in **(B),** and percentage of dead cells was determined by trypan blue exclusion assay. Graph depicts mean ± SE of the percentage of dead cells with and without IFNγ (n=3). **, $p<0.005$, ns, non-significant. **(D)** Heatmap of SILAC-measured expression of 35 upregulate ISGs in IFNγ treated LNCaP-JAK1 (25 ng/ml, 14 h) relative to untreated LNCaP-JAK1 cells.ISG identificantion and values were as in Fig. 7B.

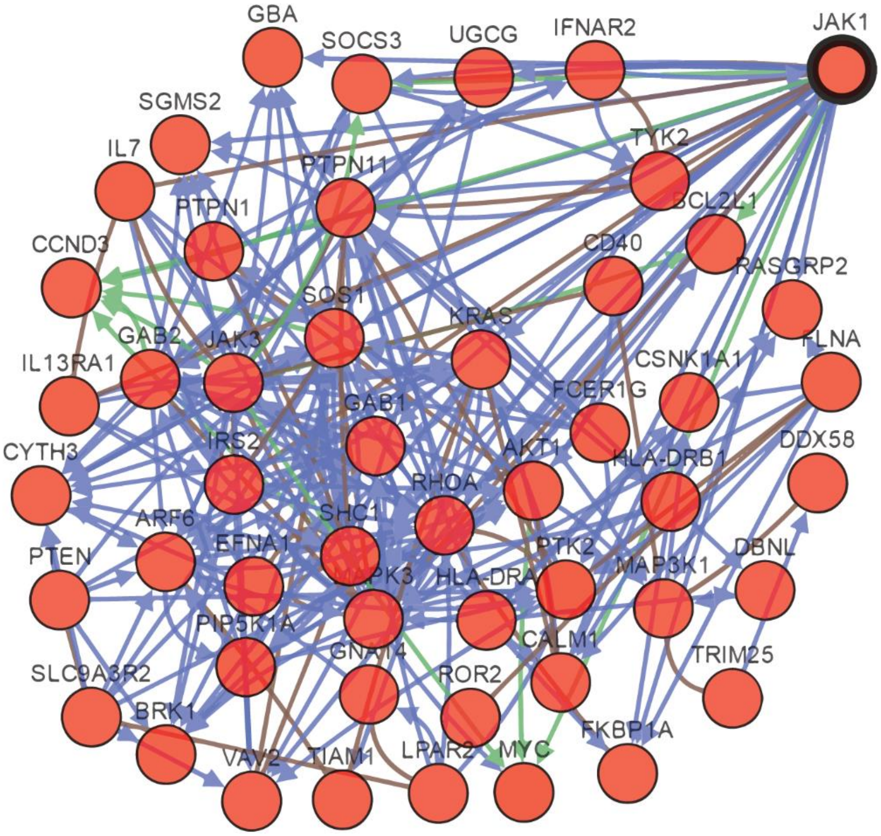

Supplementary Figure 4. Network of genes jointly altered with JAK1 in patients with metastatic PCa. Network depicts 50 jointly altered genes in more than 50% of PCa samples (*64*). Arrows indicate change of state (blue), control of expression (green), or protein interactions (brown)

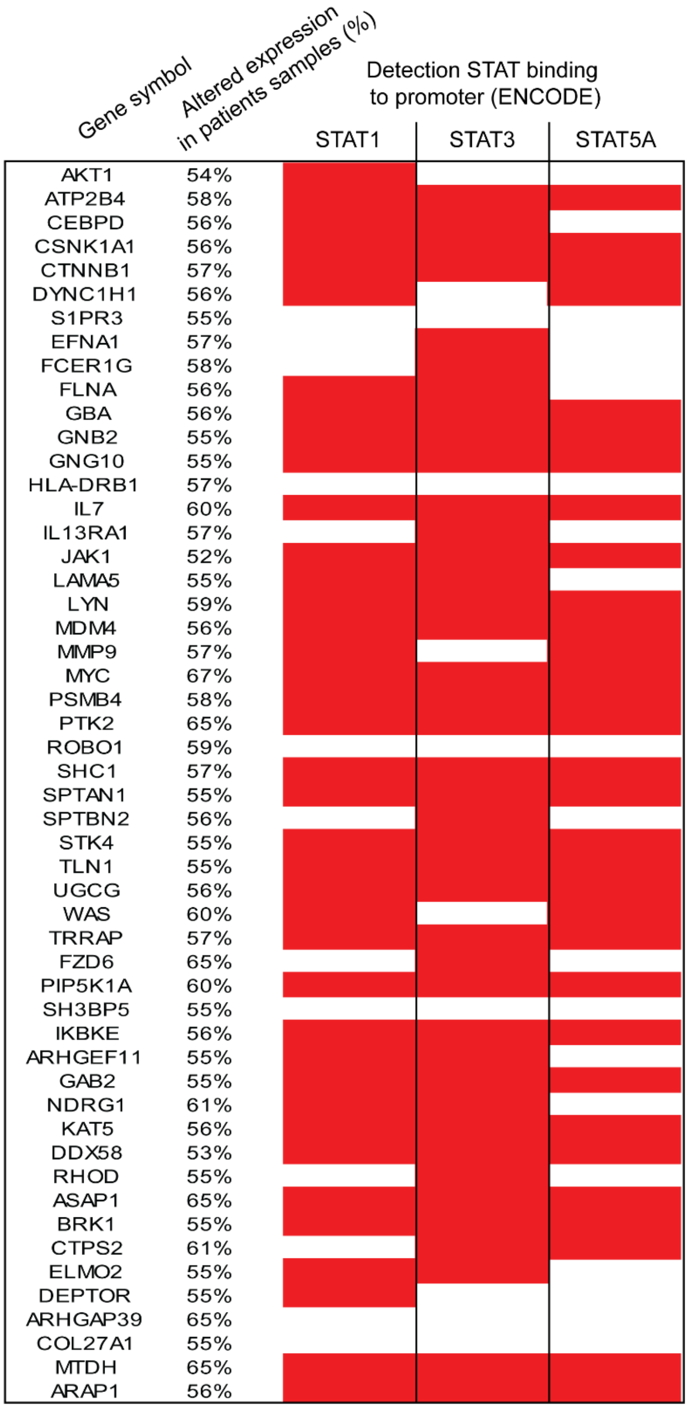

Supplementary Figure 5. STAT-target genes are jointly upregulated in metastatic PCa patient samples. Table showing binding (red) of STAT1, STAT3 and STAT5a to promoters of the indicated genes, as detected by chromatin immunoprecipitation- DNA sequencing (ChIP-seq),. Genes shown are from the network oresented in Fig. S3. Chip-seq data were retrieved from the Harmonizome web server (*83*).
